# Limitations of rapid diagnostic tests in malaria surveys in areas with varied transmission intensity in Uganda 2017-2019: Implications for selection and use of HRP2 RDTs

**DOI:** 10.1371/journal.pone.0244457

**Published:** 2020-12-31

**Authors:** Agaba B. Bosco, Joaniter I. Nankabirwa, Adoke Yeka, Sam Nsobya, Karryn Gresty, Karen Anderson, Paul Mbaka, Christiane Prosser, David Smith, Jimmy Opigo, Rhoda Namubiru, Emmanuel Arinaitwe, John Kissa, Samuel Gonahasa, Sungho Won, Bora Lee, Chae Seung Lim, Charles Karamagi, Qin Cheng, Joan K. Nakayaga, Moses R. Kamya

**Affiliations:** 1 College of Health Sciences, Makerere University, Kampala, Uganda; 2 National Malaria Control Division, Kampala, Uganda; 3 Infectious Diseases Research Collaboration, Kampala, Uganda; 4 Australian Defence Force Malaria and Infectious Disease Institute, Queensland, Australia; 5 The Army Malaria Institute Laboratory, QIMR Berghofer Medical Research Institute, Kampala, Uganda; 6 World Health Organization Country Office, Kampala, Uganda; 7 National Health Information Division, Ministry of Health, Kampala, Uganda; 8 Department of Public Health Sciences, Seoul National University, Seoul, S. Korea; 9 Department of Laboratory Medicine, College of Health Sciences, Korea University, Seoul, S. Korea; Instituto Rene Rachou, BRAZIL

## Abstract

**Background:**

*Plasmodium falciparum* histidine-rich protein 2 (HRP2)-based rapid diagnostic tests (RDTs) are exclusively recommended for malaria diagnosis in Uganda; however, their functionality can be affected by parasite-related factors that have not been investigated in field settings.

**Methods:**

Using a cross-sectional design, we analysed 219 RDT-/microscopy+ and 140 RDT+/microscopy+ dried blood spots obtained from symptomatic children aged 2–10 years from 48 districts in Uganda between 2017 and 2019. We aimed to investigate parasite-related factors contributing to false RDT results by molecular characterization of parasite isolates. ArcGIS software was used to map the geographical distribution of parasites. Statistical analysis was performed using chi-square or Fisher’s exact tests, with P ≤ 0.05 indicating significance. Odds ratios (ORs) were used to assess associations, while logistic regression was performed to explore possible factors associated with false RDT results.

**Results:**

The presence of parasite DNA was confirmed in 92.5% (332/359) of the blood samples. The levels of agreement between the HRP2 RDT and PCR assay results in the (RDT+/microscopy+) and (RDT-/microscopy+) sample subsets were 97.8% (137/140) and 10.9% (24/219), respectively. Factors associated with false-negative RDT results in the (RDT-/microscopy+) samples were parasite density (<1,000/μl), *pfhrp2/3* gene deletion and non-*P*. *falciparum* species (aOR 2.65, 95% CI: 1.62–4.38, P = 0.001; aOR 4.4, 95% CI 1.72–13.66, P = 0.004; and aOR 18.65, 95% CI: 5.3–38.7, P = 0.001, respectively). Overall, gene deletion and non-*P*. *falciparum* species contributed to 12.3% (24/195) and 19.0% (37/195) of false-negative RDT results, respectively. Of the false-negative RDTs results, 80.0% (156/195) were from subjects with low-density infections (< 25 parasites per 200 WBCs or <1,000/μl).

**Conclusion:**

This is the first evaluation and report of the contributions of *pfhrp2/3* gene deletion, non-*P*. *falciparum* species, and low-density infections to false-negative RDT results under field conditions in Uganda. In view of these findings, the use of HRP2 RDTs should be reconsidered; possibly, switching to combination RDTs that target alternative antigens, particularly in affected areas, may be beneficial. Future evaluations should consider larger and more representative surveys covering other regions of Uganda.

## Background

In 2019, the World Health Organization (WHO) estimated that there were 229 million cases of and 409,000 deaths due to malaria globally. The WHO African region accounts for a disproportionately high share of the global burden (94% of malaria cases in 2019 alone) [1,2]. Nearly all malaria cases in the WHO African region are caused by *Plasmodium falciparum*. Uganda is ranked among the top six countries with the highest malaria burdens [1–3]. Malaria remains a major public health problem in Uganda, with 16 million cases annually, accounting for 30% of outpatient visits to health facilities (HF), 14–20% of hospital admissions and 8–10% of inpatient deaths [4–6]. Although the epidemiology of malaria varies, it is endemic throughout the whole country, and transmission occurs year-round. *P*. *falciparum* accounts for >95% of malaria infections in Uganda [6–11]. Efforts to reduce the burden of malaria have included the use of long-lasting insecticidal nets (LLINs), indoor residual spraying (IRS) of insecticides, intermittent preventive therapy (IPT) and diagnosis and treatment of cases [10,12].

Case management that involves testing and treatment is a major intervention for malaria control in Uganda. The WHO recommends parasitological confirmation of malaria in all suspected malaria cases prior to the administration of antimalarial treatment [13]. Uganda adopted the policy shift from a clinical to parasite-based diagnosis with microscopy or rapid diagnostic tests (RDTs) in 2011 [10]. Blood smear microscopy is the gold standard for malaria diagnosis because it is inexpensive to perform and is able to differentiate malaria species and quantify parasites. However, microscopy requires well-trained, competent microscopists and functional infrastructure as well as effective quality control (QC) and quality assurance (QA) systems. RDTs utilise monoclonal antibodies that are impregnated on a test strip and directed against the target parasite antigen to detect malaria antigens in a small amount of blood. RDTs are increasingly favoured for malaria diagnostic confirmation because they require no capital investment or electricity, are simple to perform and are easy to interpret [10]. Due to the predominance of *P*. *falciparum* which accounts for >95%, the country’s malaria diagnosis policy recommends the use of HRP2 antigen-based RDTs as the most effective type of RDT [10,11]. Since the introduction of RDTs in the late 2000s, over 900 million RDTs have been used for malaria testing in Uganda, all of which were HRP2-based and targeted *P*. *falciparum*. As a result, HRP2-specific RDTs currently account for >80% of the total malaria tests in Uganda [6,10]. However, with the changing epidemiology of malaria as countries advance towards elimination, highly sensitive diagnostic tools that detect low-density parasite infections and sub-patent infections will be required. Currently, only nucleic acid amplification tests (NAATs) are sufficiently sensitive to detect these low-density infections. However, this method is limited to well-equipped laboratory settings due to its inherent complexity and need for laboratory equipment [14,15].

Malaria RDTs are known to capture at least three target antigens: lactate dehydrogenase (LDH), *Plasmodium falciparum* histidine-rich protein 2 (PfHRP2) and pan-plasmodial aldolase. HRP2 RDTs are the most sensitive for parasite detection and are heat-stable under field conditions compared to the other antigen tests [16,17]. However, HRP2 RDTs have limitations, as their performance has been shown to be affected by product quality and parasite-related factors such as *pfhrp2/3* gene deletion, non-*P*. *falciparum* species and prozone effects that lead to false-negative RDTs [18–23]. The presence of *pfhrp2* and *pfhrp3* gene deletions in *P*. *falciparum* parasite populations has been reported in Uganda [24,25] and other malaria endemic countries in sub-Saharan Africa [18,19,21,26–31]. Additionally, there is an increasing prevalence of non-*P*. *falciparum* species in Uganda [5,11]. *P*. *falciparum* parasites that lack the *pfhrp2*/*3* genes and non-*P*. *falciparum* species are not detected by HRP2 RDTs and may contribute to false-negative RDT results, leading to a reduction in the effectiveness of these tests [19,21,32,33]. Evidence of the possible contributions of parasite gene deletions, non-*P*. *falciparum* species and low-density infections to false-negative HRP2 RDT results in Uganda is limited. As Uganda advances towards malaria elimination, it is important to ensure that all malaria infections are detected by effective diagnostic tools and treated promptly to enhance case management and surveillance-based interventions. In this study, we assessed the possible factors contributing to false-negative HRP2-based RDTs in blood samples collected from 48 districts in Uganda.

## Methods

### Study design and setting

This was a cross-sectional study that analysed dried blood spots collected during previous malaria surveys of symptomatic individuals in 48 districts of Uganda between 2017 and 2019 [34–36]. The surveys were designed to evaluate the effect of different types of LLINs on parasite prevalence, covering nearly half of the country and a wide range of epidemiological settings [35,36]. Malaria is endemic in 95% of the country, and transmission occurs throughout the year, with two peak transmission seasons in June—July and November—December [35–37]. The parasite surveys were conducted at 6-month intervals that coincided with the two peak transmission seasons.

### Study population, participant selection and data collection

**Fig 1:**
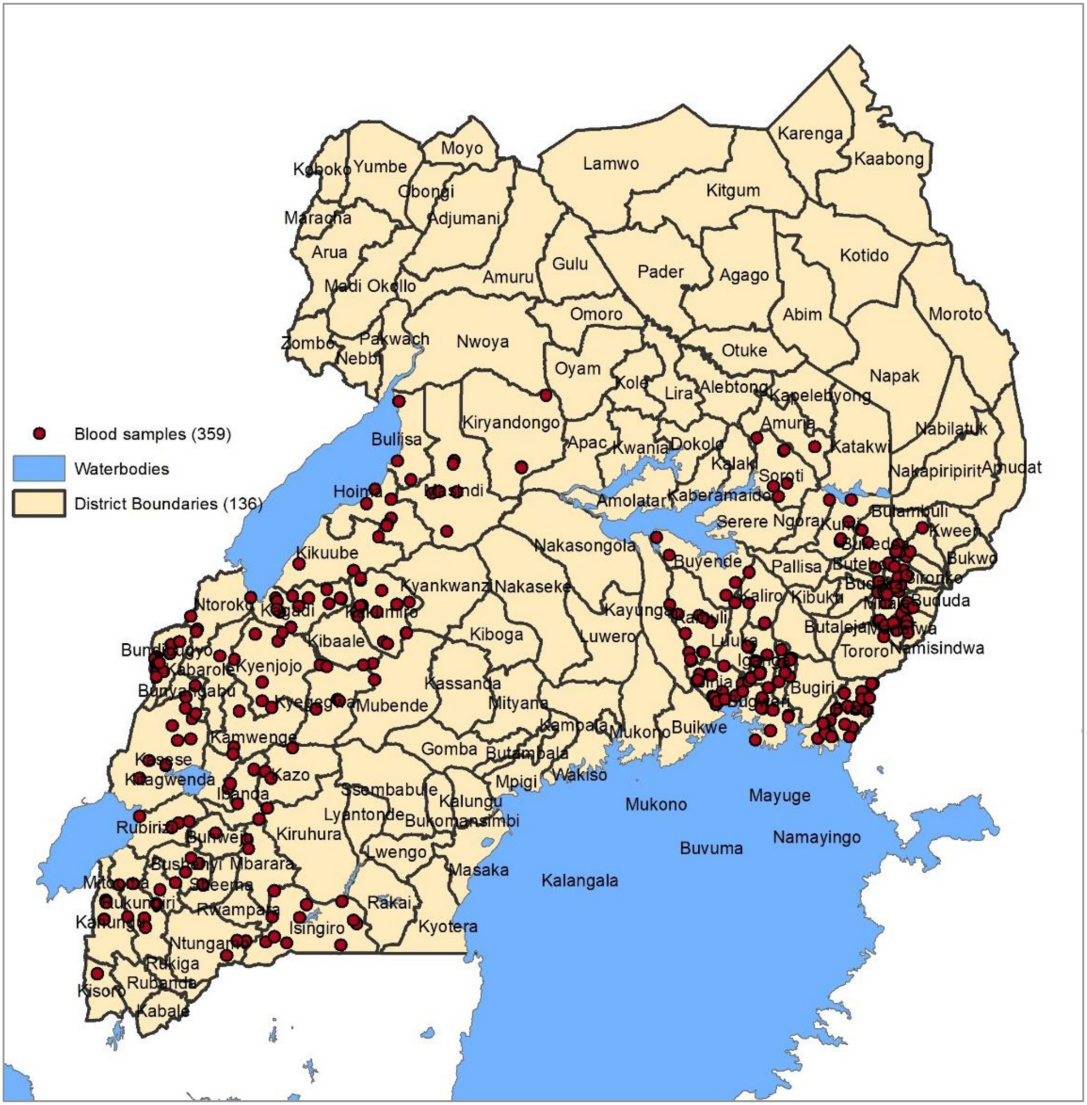
Geographical distribution of DBS samples across the study areas. The red dots represent the DBS samples.

Details of sampling, participant selection and enrolment in the previous malaria surveys in which the DBS were obtained have been described and published elsewhere [35,36]. In brief, a total of 104 clusters (health sub-districts) across 48 districts were selected and randomized to receive different LLINs. Fifty (n = 50) households were randomly selected from each cluster to participate in the study. In the selected households, children aged 2–10 years were assessed for the presence of fever (based on an axillary temperature of >37.5°C) before enrolment. Enrolled children were tested for malaria using HRP2 RDTs, and diagnosis was confirmed by microscopy [35,36]. Additionally, dried blood spots (DBS) were collected and stored for molecular testing of parasites. Written consent was obtained from the parents/guardians of the children, and assent was obtained from the children aged 8 years and above prior to commencement of the study procedures. This study utilised the data and DBS collected during malaria surveys. A GIS map of sites where the DBS samples were collected is indicated in Fig 1.

### Selection of dried blood spots

**Fig 2:**
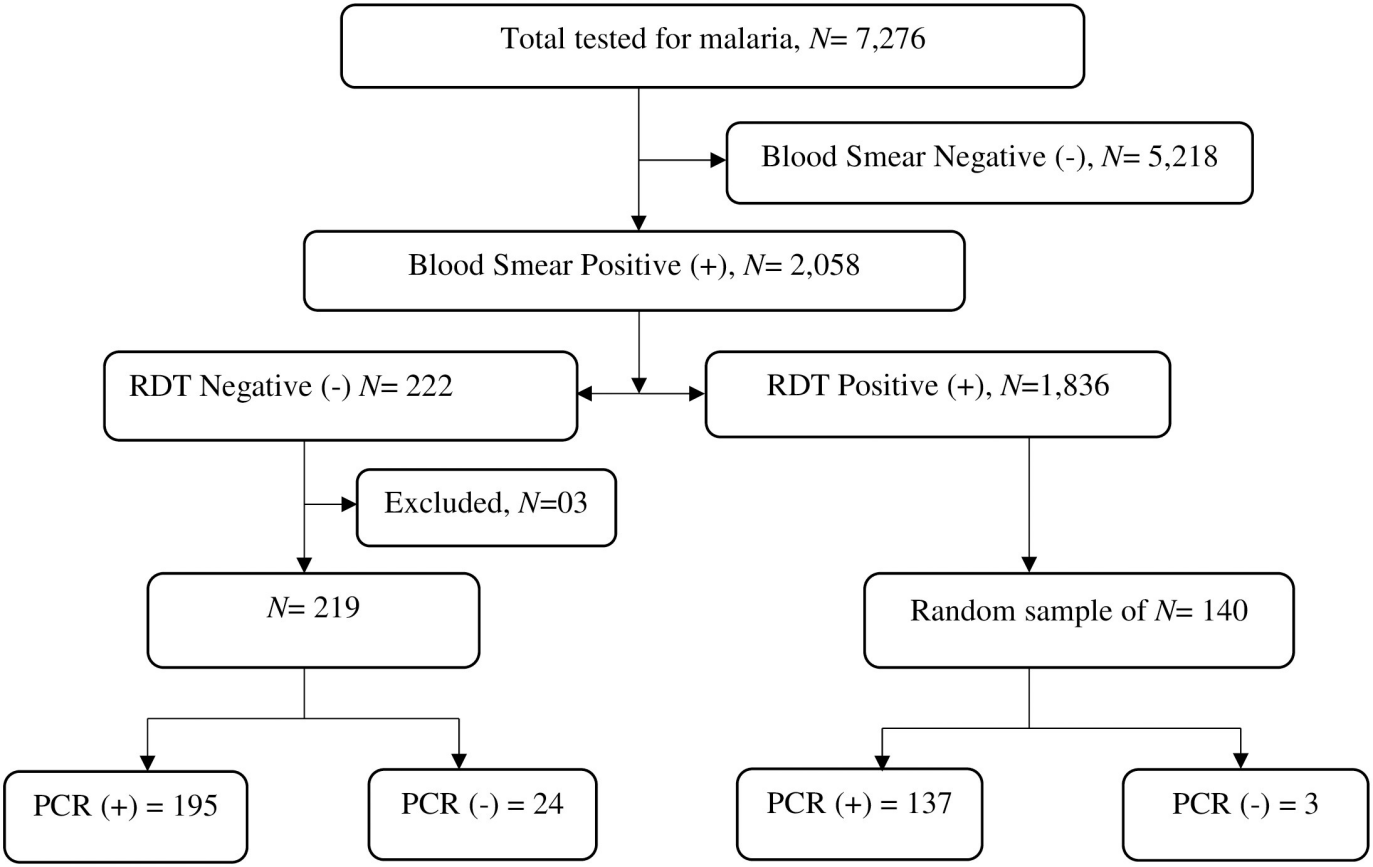
Study profile. Shows how the (RDT-/microscopy+) and (RDT+/microscopy+) DBS samples were selected. (RDT-/microscopy+) means samples that were RDT negative but microscopy positive for malaria while (RDT+/microscopy+) are samples that were positive on both RDTs and microscopy.

A total of 7,276 symptomatic participants were tested for malaria in previous surveys using HRP2 RDTs and blood smear microscopy, of whom 2,058 (28.3%) had positive blood smears according to microscopy. In addition, DBS were collected for all samples. Of the 2,058 positive samples, 10.8% (222/2058) had a negative HRP2 RDT despite a positive blood smear (RDT-/microscopy+). Out of these 222 samples, three (3) samples were excluded due to contamination, leaving 219 for final analysis. In this study, we conducted molecular analyses for all 219 (RDT-/microscopy+) samples and a randomly selected subset of (RDT+/microscopy+) samples. In brief, from a list of all 1,836 (RDT+/microscopy+) samples, simple random sampling was used to select 140 DBS for molecular analysis. PCR was performed on all the selected samples to confirm the presence of parasite DNA and species determination. Details of the sample selection procedure are indicated in the study profile (Fig 2).

### Laboratory analysis

#### Rapid diagnostic tests (RDTs)

The HRP2 RDT results were available in the previous malaria survey database. During the surveys, HRP2-based *P*. *falciparum*-specific RDTs (SD Bioline Malaria Ag Pf 05FK120; Standard Diagnostics, Gyeonhhi-do, South Korea) were used to test for malaria in febrile patients with a history of fever (based on an axillary temperature of >37.5°C). The test is designed to detect only *P*. *falciparum* infections. RDTs were performed according to the manufacturer’s instructions.

#### Blood smear microscopy

All blood smear microscopy results were obtained from the previous survey database. In brief, blood smears were stained with 2% Giemsa for 30 minutes. Each blood smear slide was read independently by two competent (level 1 WHO certified reader) laboratory scientists. The slide readers were blinded to each other’s results and were not aware of participants’ RDT results. Thick blood smears were evaluated for the presence of parasites (asexual forms) and gametocytes following the standard WHO methodology [38]. Parasitaemia was determined by counting the number of parasites per 200 or 500 WBCs for low-density infections on thick smears (assuming a standard of 8,000 WBCs per μl in accordance with WHO methods) [38]. Smears were considered negative if no parasites were observed in 200 oil-immersion fields (1000X) in a thick blood film. For quality assurance purposes, 20% of blood smears were retrieved and crosschecked for the presence of parasites.

#### Parasite DNA extraction

The dried blood spots (DBS) were shipped to the Australian Defence Forces Malaria and Infectious Disease Institute (ADFMIDI) Brisbane, Queensland, Australia, where all molecular testing was conducted. Details of DNA extraction by QIAamp DNA Mini Kits (QIAGEN, Crawley, UK) and the QIAcube robotic platform (QIAGEN, Crawley, UK) have been described and published elsewhere [21,25,32,33,39,40]. In brief, from each DBS sample, three discs of dried blood were punched into 1.5 mL microfuge tubes. DNA was extracted using QIAamp DNA Mini Kits and the QIAcube robotic platform (QIAGEN, Crawley, UK) according to the manufacturer’s instructions. Samples were eluted to a volume of 100 μL with AE buffer. *P*. *falciparum* positive control DBS spots were processed and run alongside the samples.

#### Confirmation of parasite DNA and *Plasmodium* speciation

The detailed procedure for the controls, primers and PCR conditions used for amplification, speciation and detection of parasite DNA has been widely described and published elsewhere [21,25,32,33,39,40]. In brief, different *Plasmodium* species in the blood samples were confirmed by amplification of the *18S ribosomal RNA* (18S rRNA) gene using multiplex PCR. The primer sequences used for PCR amplification of the different species are indicated in S1 Table. The presence of *P*. *falciparum* infection was further confirmed by *P*. *falciparum*-specific PCR and amplification of the MSP1 and MSP2 single-copy genes. Gel electrophoresis using 2% agarose was used to confirm the presence of bands.

#### Amplification of *pfhrp2* and *pfhrp3* genes

The detailed procedure, primers used and PCR conditions used for amplification and detection of the *pfhrp2* and *pfhrp3* genes are well described and published elsewhere [21,25,32,33,41]. In brief, all samples that were confirmed as *P*. *falciparum-*positive and in which *Merozoite Surface Protein 1* (MSP1) and *Merozoite Surface Protein 2* (MSP2) genes were detected, the exon 1 and exon 2 of the *pfhrp2* and *pfhrp3* genes were amplified to investigate the presence or absence of the *pfhrp2* and *pfhrp3* genes. The primer sequences used for the amplification of *pfhrp2* and *pfhrp3* exon 1 and exon 2 are indicated in the supplemental data (Table 2). PCR controls using laboratory lines DD2, 3BD5, HB3 and 3D7 with known *pfhrp2/3* status and human negative controls were included in each PCR run. PCR runs were considered valid only if all controls were amplified and resulted in bands of expected sizes on gel electrophoresis. In all cases, samples were considered gene-deleted if they were positive for *P*. *falciparum* DNA on PCR and the presence of the MSP1 and MSP2 single-copy genes were confirmed but exon 1 or exon 2 of the *pfhrp2* or *pfhrp3* genes failed to amplify despite amplification in assay controls.

#### Quality control

As part of quality control, blood smear microscopy was performed in a blinded manner by level 1 WHO-certified microscopists. In addition, a random sample of 20% of the slides was re-read by two level one WHO-certified microscopists. A third level 1 expert resolved any discrepancies (difference in parasite counts between two microscopy readings >20%). All three slide readers were independent of the process and from an external laboratory. The research laboratory in Australia, where molecular analysis of the samples was performed, is a WHO collaborating centre for malaria, a member of the WHO *pfhrp2* and *pfhrp3* gene deletion detection laboratory network and participates in the WHO NAAT external quality assurance programme.

#### Ethical approval

The study was approved by the Makerere University School of Medicine Research Ethics Committee (#REC REF 2017–111), the Uganda National Council of Science and Technology (Ref No: HS271ES), and the Australian Department of Defence and Veterans’ Affairs Human Research Ethics Committee (DDVA HREC 096–18). In the primary surveys in which the samples were collected, participants were enrolled after providing written informed consent. Only samples from participants who provided consent for future use of biological samples were selected for this analysis.

#### Data management and statistical analysis

As part of data management, demographics and predictor variables linked to the blood samples were extracted from the previous survey database. All data were entered and managed in Excel before they were exported to STATA for analysis. Data quality checks were performed to check for and correct any inconsistencies using pivot tables in Excel. ArcGIS software version 10.8 (Environmental Systems Research Institute (Esri), California U.S.) was used to map the sites where the DBS were collected and where the different parasite species occurred. Data analysis was performed with STATA version 14 (StataCorp LP, College Station, TX). Statistical testing was performed with chi-square or Fisher’s exact tests, with P ≤ 0.05 indicating significance. The odds ratio was used to evaluate the association, while logistic regression analysis was performed to explore the factors associated with false RDT results.

## Results

### Baseline characteristic of the samples

In this study, we conducted molecular analysis of 359 DBS samples that were collected from symptomatic individuals in previous malaria surveys to investigate parasite-related factors contributing to false-negative HRP2 RDTs in Uganda. Overall, 92.5% of the samples (332/359) contained parasite DNA confirmed by PCR. The majority of the DBS samples came from participants who were aged ≥5 years (58.8%), male (50.7%) and mostly from the eastern region of Uganda (51.3%). Most had a parasite density ≥1,000/μl (59.6%). *P*. *falciparum* was the most predominant species (83.5%), followed by *P*. *malariae* (6.4%) and *P*. *ovale* (1.9%). Molecular characterization by *pfhrp2/3*-specific PCR showed that 24 isolates in the (RDT-/microscopy+) subset and 5 in the (RDT+/microscopy+) subset were infected with parasites with *pfhrp2* and *pfhrp3* gene deletions. The 24 gene-deleted isolates in the RDT-/microscopy+ subset included 9 isolates with *pfhrp2* single deletions (pfhrp2-), 5 isolates with *pfhrp3* single deletions (*pfhrp3*-) and 10 isolates with *pfhrp*2/3 double deletions (*pfhrp2*-/*pfhrp3-*). The detailed characteristics of the samples are indicated in Table 1.

**Table 1:** Baseline characteristics of the samples.

Variable	Frequency (n)	Proportion (%)
**Age (year)**		
<5	148	41.2
≥5	211	58.8
**Sex**		
Male	182	50.7
Female	177	49.3
**Parasite density (μL)**		
<1000	145	40.4
≥1000	214	59.6
**Region**		
Eastern	184	51.3
Western	175	48.7
**Endemicity**		
Low	249	69.4
Moderate	110	30.6
**Parasitaemia (PCR)**		
Positive	332	92.5
Negative	27	7.5
**Gene deletion**		
*pfhrp* 2/3 deletion (any)	29	8.1
No deletion	330	91.9
**Parasite species**		
*P*. *falciparum*	300	83.5
*Non-P*. *falciparum*	39	10.9
**Species composition**		
*P*. *malariae*	25	6.4
*P*. *falciparum*	295	82.2
*P*. *ovale*	7	1.9
*P*. *falciparum/malariae*	2	0.6
*P*. *malariae/ovale*	2	0.6
*P*. *falciparum/vivax*	2	0.6
*P*. *falciparum/ovale*	1	0.3

Frequency is the number of observations, while proportion is the percentage of those observations. <1,000 and ≥1,000 indicate parasite densities of less than 1,000 and more than 1,000 parasites per microlitre of blood, respectively. Mixed infections are samples infected with more than one *Plasmodium* species.

**Fig 3:**
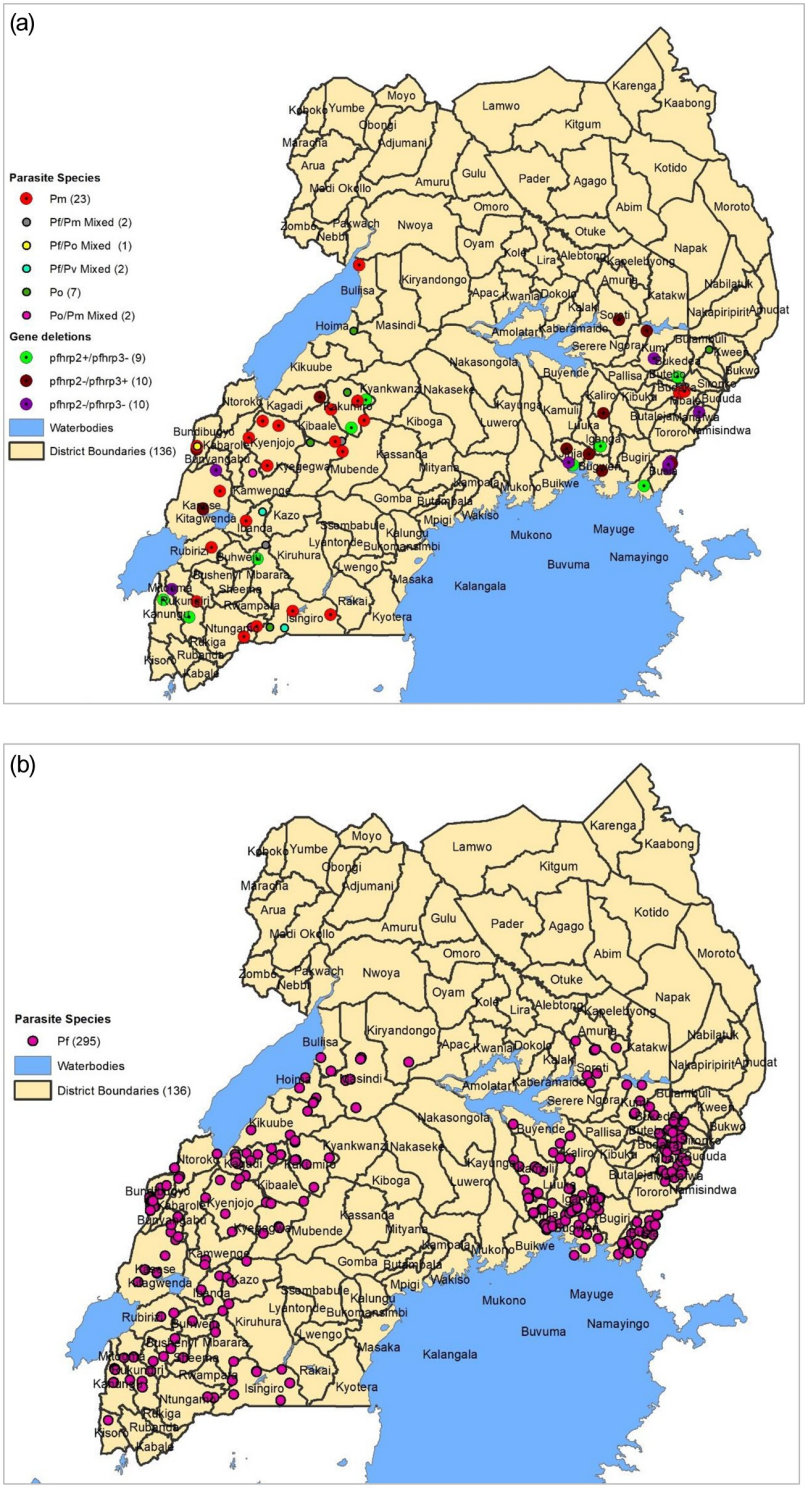
3a and 3b: Each circle represents one parasite isolate. Black lines represent administrative boundaries.

Based on multiplex PCR, all the four *Plasmodium* parasite species (*P*. *falciparum*, *P*. *malariae*, *P*. *ovale and P*. *vivax*) were encountered in the samples. Using GIS, the different parasite species and gene deleted isolates observed in the samples were mapped to determine their distribution pattern. Overall, the non-*P*. *falciparum* species were distributed across both regions (Fig 3a); however, *P*. *falciparum* was the most prevalent species (Fig 3b).

### Summary of RDT, microscopy and PCR results of the DBS samples

The levels of agreement between the HRP2 RDT and PCR results in the (RDT+/microscopy+) and (RDT-/microscopy+) sample subsets were 97.9% (137/140) and 10.9% (24/219), respectively. This observation suggests that 195 (89.0%) of 219 (RDT-/microscopy+) samples falsely registered as negative on HRP2 RDTs (Table 2). An important observation is that out of the 195 false-negative RDT results identified, non-*P*. *falciparum* species and *pfhrp2/3* gene deletions contributed 19.0% (37/195) and 12.3% (24/195), respectively. All 195 samples falsely registered as negative on RDTs were from subjects with low-density infections [median (IQR); 420 (112–880)]. On the other hand, the levels of agreement between blood smear microscopy and PCR in the (RDT+/microscopy+) and (RDT-/microscopy+) subsets were 97.9% (137/140) and 89.0% (195/219), respectively. This suggests that 10.9% (24/219) of the (RDT-/microscopy+) samples falsely registered as positive on blood smear microscopy. Reports of false-positive blood smears from the field are not uncommon, particularly in resource-limited settings where the functionality of malaria microscopy is highly compromised by inadequate infrastructure, skills and logistics [38]. The presence of extremely low parasitaemia and non-*P*. *falciparum* infections, particularly among (RDT-/microscopy+) samples, may have posed detection challenges to slide readers.

**Table 2:** Summary of DBS results for RDTs, microscopy and PCR.

Variable	PCR (RDT-/microscopy+) *n* = 219)		PCR (RDT+/microscopy+) *n* = 140)	
	Positive	Negative	Positive	Negative
**RDT**				
Positive	0 (0%)	0 (0%)	137 (97.9%)	3 (2.1%)
Negative	195 (54.3%)	24 (10.9%)	0 (0%)	0 (0%)
**Microscopy**				
Positive	195 (89.0%)	24 (10.9%)	137 (97.9%)	3 (2.1%)
Negative	0 (0%)	0 (0%)	0 (0%)	0 (0%)

(RDT-/microscopy**+)** are samples that were negative on the RDT but positive on blood smear microscopy, RDT+/microscopy+ are samples that were positive on both the RDT and blood smear microscopy.

### Factors associated with false-negative RDTs

Additional data were obtained to assess factors possibly contributing to false-negative RDTs. We performed two-level logistic regression analysis. First, we fit and ran a model with all the variables, and then using backward model selection, we retained only those factors that were significant (P<0.05) (Table 3). In the final regression model, the factors associated with false-negative RDT results were *pfhrp2/3* gene deletion, non-*P*. *falciparum* species and low parasite density (aOR = 4.4 (95% CI: 1.7–13.7) P = 0.004; aOR = 18.7 (95% CI: 5.5–118.7) P = 0.001; and aOR = 2.7 (95% CI: 1.6–4.4) P = 0.001, respectively) (Table 3).

**Table 3:** Factors associated with false-negative RDT results using PCR as a reference.

Variable	RDT-/PCR+/False-negative	Univariable		Multivariable	
	n (%)	OR (95% CI)	p-value	aOR (95% CI)	p-value
**Age (year)**					
≥5	108 (55.7)	1 (Reference)	0.179	1 (Reference)	
<5	87 (63.0)	1.36 (0.87–2.13)		1.35 (0.83–2.19)	0.224
**Parasite density (μL)**					
≥1000	105 (51.0)	1 (Reference)		1 (Reference)	
<1000	90 (71.4)	2.4 (1.51–3.89)	0.001	2.65(1.62–4.38)	0.001
**Gene deletion**					
No deletion	171 (56.4)	1 (Reference)		1 (Reference)	
*pfhrp 2/3* deletion	24 (82.8)	3.71 (1.49–11.23)	0.01	4.4 (1.72–13.66)	0.004
**Parasite species**					
*P*. *falciparum*	165 (55.0)	1 (Reference)		1 (Reference)	
Non-*P*. *falciparum*	37.0 (93.9)	12.76 (3.77–79.7)	0.001	18.65 (5.3–118.71)	0.001
**Endemicity**					
Moderate	63 (61.2)	1 (Reference)		1 (Reference)	
Low	132 (57.6)	1.16 (0.72–1.87)	0.547	1.19 (0.63–2.24)	0.595
**Region**					
Western	95 (57.6)	1 (Reference)		1 (Reference)	
Eastern	100 (59.9)	0.91 (0.59–1.41)	0.67	0.83 (0.45–1.5)	0.527

OR = unadjusted odds ratio, aOR = adjusted odds ratio.

## Discussion

### Summary of findings

HRP2-based RDTs are currently the most commonly used tools for malaria diagnosis in Uganda and other parts of sub-Saharan Africa, where *P*. *falciparum* is the predominant parasite species [1,3,10]. However, many factors can affect the effectiveness of RDTs as malaria diagnostic tools and require periodic monitoring [18–21,31,33]. In Uganda, there are unpublished field reports on and concerns about the occurrence of false-negative RDT results; however, previous investigations focused on only the products, systems and user-related factors. In this study, we used molecular assays to assess the parasite-related factors contributing to false-negative RDTs using 359 (RDT-/microscopy+) and (RDT+/microscopy+) DBS samples collected from different malaria epidemiological settings in 48 districts in Uganda. Overall, the presence of parasite DNA was confirmed in 195 DBS that had registered as negative on HRP2 RDTs. The low level of agreement between the HRP2 RDT and PCR results could be attributed mainly to the reduced ability of HRP2 RDTs to detect antigens at low parasite densities and parasites with HRP2 gene deletions and the inability to detect non-*falciparum* species observed in these samples.

### Low parasite densities

We investigated parasite densities in relation to false-negative RDT results. Parasite densities were generally lowest in the RDT-/PCR+ samples. We showed that 71.4% (95% CI: 62.7–79.1) of low-density samples were in the RDT-/PCR+ group, in which all the false-negative RDTs occurred (p = 0.001). This correlated with the multiple logistic regression analysis that showed that samples with low parasite densities were more likely to produce false-negative RDT results (aOR = 2.65, 95% CI (1.62–4.38), P = 0.001). This observation can be explained by the fact that there are inadequate or undetectable levels of HRP2 antigens available in low-parasite density samples, as shown in other studies [42–44]. The implication of this observation is that as Uganda advances towards malaria elimination, the burden due to extremely low parasitaemia/a low-density and sub-microscopic parasite load is likely to increase, requiring more appropriate diagnostic tools.

### HRP2 deletions

When further investigated by molecular characterization, 24 out of the 195 false-negative RDTs were blood samples infected with *P*. *falciparum* parasites that lacked the *pfhrp2 (n* = 9*)*, *pfhrp3* (*n* = 5) or both the *pfhrp2* and *pfhrp3* (*n* = 10) genes. Importantly, all parasites with both *pfhrp2* and *pfhrp3* gene deletions were identified in the RDT-/PCR+ group, in which all the false-negative RDT results occurred. These parasites do not express HRP2/3 antigens and thus cannot be detected by HRP2-based RDTs [20,31–33]. In this study, gene deletions accounted for 12.3% (24/195) of the total false-negative RDT results in the (RDT-/microscopy+) samples. However, an interesting observation in this study was the occurrence of five parasite strains that carried gene deletions (one with *pfhrp2* deletion and four with *pfhrp3* deletion) in the RDT+/PCR+ group that were originally reported as RDT+. No parasite in this group carried the double *pfhrp2/3* deletion. The occurrence of gene deletion in the RDT-/PCR+ samples suggests that gene deletion is a contributing factor to false-negative RDTs, particularly in areas where the isolates were collected. This was consistent with the results of the logistic regression analysis, which showed a significant relationship between gene deletion and false-negative RDT results (aOR = 4.4, 95% CI (1.7–13.7), P = 0.004). This observation suggests that parasite gene deletion was one of the contributing factors to false-negative RDTs in these samples and supports the results of previous studies that found a similar association [18,19,21,29,32]. Gene deletions have been shown to cause false-negative RDT results in the Amazon region, where they were first identified in clinical samples, Eritrea, Mali, Rwanda and India [18,19,21,32,45,46]. Many studies conducted in the Amazon and Africa have indicated that gene-deleted *P*. *falciparum* parasites lack the *pfhrp2/3* genes and therefore lack the HRP2 antigen epitopes and repeat sequences that are essential for antibody binding [18,19,21,29,32]. Studies have suggested the possibility of the evolution of gene-deleted parasites by a genetic event due to selective pressure resulting from long-term use of HRP2-based RDTs [21]. Long-term use of HRP2 RDTs has been shown to cause selective pressure that leads to the emergence, multiplication and spread of *pfhrp2/3* gene-deleted parasites [21,31]. In Uganda, HRP2 RDTs were introduced into the testing programme in 2011 and have been used nationwide to scale up parasite-based diagnosis since 2010 [10]. In Eritrea, studies suggested that clonal expansion of *pfhrp2/3*-deleted parasites may have been caused by selective pressure due to long-term use of HRP2-based RDTs. This explanation is supported by recent mathematical modelling that showed that the exclusive use of HRP2-based RDTs exerts strong selection pressure for *pfhrp2*-negative parasites in the population that can potentially spread [14,21,47]. This suggests that parasites with deleted genes are likely to be among the contributors to false-negative HRP2 RDTs in Uganda, particularly in areas that have been mapped.

### Non-*P*. *falciparum* species

We performed multiplex PCR and showed that 37 out of the 195 false-negative RDTs were samples infected with non-*P*. *falciparum* species, Pm (n = 25), Po (n = 5) and mixed infections (n = 7). Non-*P*. *falciparum* species do not express HRP2 protein antigen and therefore are not detected by HRP2-based RDTs. In this study, non-*P*. *falciparum* species contributed to 19.0% (37/195) of false-negative RDTs. The observed presence of non*-P*. *falciparum* species in these samples is consistent with the results of a recent 2019 national malaria indicator survey that reported an increase in non-*falciparum* species, particularly *P*. *malariae* and *P*. *ovale*, in Uganda [5]. Interestingly, over 90.0% of non-*P*. *falciparum* species observed in this study occurred in the samples that had tested negative by RDTs in the (RDT-/PCR+) group, in which all the false negatives occurred. This corelated with the logistic regression analysis results that showed an association between non-*P*. *falciparum* species and false-negative RDTs (aOR = 18.7, 95% CI (5.3–118.71), P = 0.001). This observation is consistent with the results of a number of studies elsewhere that showed the occurrence of false-negative HRP2 RDTs in non-*P*. *falciparum* clinical samples [22]. The increase in the prevalence of non-*P*. *falciparum* species in Uganda suggests that combination RDTs that target alternative antigens may be more appropriate for use in case management and surveillance in these settings [48].

### Other causes of false-negative HRP2 RDTs

Many factors can affect the functionality of RDTs; these factors include product design, transport and storage conditions, parasite-related factors and operator-related factors [33,45,49]. Prior to this study, there was limited evidence of parasite-related factors contributing to false-negative RDTs in real field settings in Uganda, as previous investigations focused on products and user-related factors. Many parasite-related factors, including *pfhrp2/3* gene deletion, have not been studied on a broad scale in many parts of Africa, and evidence remains limited [31,33]. Failure of the parasite to express the HRP2 target antigen or alteration in the HRP2 protein sequence has been shown to affect the efficacy of RDTs [50–53]. Variation in the pattern and sequence of histidine repeat tandems and the number, frequency and composition of amino acids within the HRP2 protein antigen are known to affect the efficacy of HRP2 RDTs [51,53]. Other known causes of false-negative RDTs include product design, transport and storage conditions and user-related factors [33,45,49]. Many endemic countries that collaborate with the WHO and manufacturers have instituted QA systems to address most of the possible causes of false RDT results related to handling and product design through centralized RDT product testing programmes [49,54,55]. Moreover, the test used in this study was a quality-assured RDT that was WHO prequalified and had passed the WHO product testing programme requirements [54,55]. Transportation, handling and storage records were all reviewed and found to be satisfactory. The users who performed the tests in the field were well-trained laboratory technicians. The above suggests that product design, handling and user-related factors were unlikely to be the major causes of the false-negative RDT results observed in these samples.

### Implication for malaria control

The study documented for the first time the contribution of non-*P*. *falciparum* species, *pfhrp2/3* gene deletions and low parasite density infections to false-negative RDT results in Uganda. These results imply that real malaria cases are frequently missed, suggesting that HRP2 RDTs are inappropriate for the diagnosis of malaria, particularly in affected areas. Failure of HRP2 RDTs to diagnose *pfhrp2/3*-deleted parasites, non-*P*. *falciparum* species and low-density infections may have implications for malaria case management and surveillance, which impacts malaria control efforts in Uganda. In this study, blood samples were collected during malaria surveys from symptomatic individuals who had fever. Individuals were screened with a HRP2 RDT and treated immediately with ACTs if they had a positive RDT result [35,36]. The implication of these findings is that individuals infected with gene-deleted parasites and *non-falciparum* species could have missed treatment due to false-negative RDT results. Untreated non-*P*. *falciparum* species and *pfhrp2/3*-deleted parasites could undergo selection pressure that favours their survival, multiplication and spread, threatening malaria control efforts [21,31,33]. The Uganda National Malaria Control Policy recommends that treatment should be given only to individuals with a confirmed parasite-based diagnosis [10,13]. In view of the fact that HRP2 RDTs account for over 85% of malaria tests in Uganda [4], treatment and surveillance strategies may need to consider issues related to gene deletions, non-*P*. *falciparum* species and low-density infections to minimize the risk of false-negative RDT results, particularly in areas in which the parasites have been recorded. The occurrence of false-negative RDTs may also affect the overall user confidence in RDTs, which may prompt a switch to presumptive treatment and clinical diagnosis, threatening malaria control gains [33].

In view of these findings and the systematic challenges limiting the functionality of microscopic malaria diagnosis, particularly in remote and peripheral health facilities [38], combination RDTs that target alternative parasite antigens, such as lactate dehydrogenase (LDH) and aldolase, are likely to be suitable alternatives. Combination RDTs that target other antigens have been evaluated and demonstrated good performance as alternative malaria diagnostic tools in field trials in Uganda and similar settings abroad [46,56–59]. Molecular-based methods, such as PCR and loop-mediated isothermal amplification (LAMP), could provide suitable alternatives; however, they are expensive and may not be feasible for use in routine surveillance and patient care [39,40,60]. As Uganda advances towards malaria elimination, false-negative RDT results due to low-parasite density infections will be a potential threat [61,62]. Efforts to address low-parasite density infections should consider the deployment of highly sensitive diagnostic tools, including nucleic acid amplification-based tests [61,63–68]. However, the current WHO guidance does not recommend the use of ultrasensitive RDTs for routine diagnosis until additional evidence becomes available [67].

### Limitations of the study

Our study was limited by the fact that the *P*. *falciparum* isolates and the DBS samples were obtained from two regions in Uganda, meaning that the status and risk of false-negative RDT results in other regions is unknown. We recommend that future studies consider sampling from a broader area to achieve national representation. We recognize that there are other factors that may contribute to false-negative RDTs that were outside the scope of this study, including variation in the composition of the *pfhrp2* repeat sequence, number of repeat types and amino acid composition of the HRP2 protein antigen [50,51,53]. However, the other possible contributors and causes of false-negative HRP2 RDTs are well studied and published [18–21,31,33,53].

### Conclusion

This was the first wide-scale investigation to analyse the contribution of low-parasite density infections, *pfhrp2/3* gene deletions and non-*P*. *falciparum* species to false-negative RDT results in real field settings in Uganda. In view of these findings, the use of HRP2 RDTs for malaria case management and surveillance may need to be reconsidered; a switch to combination RDTs that target alternative antigens, particularly in affected areas, may be necessary. Future evaluations of false-negative HRP2 RDT results should include larger and more representative surveys covering other regions of Uganda.

## Supporting information

S1 Fig (DOCX)

S1 Table Parasite DNA amplification: Primer sequences. (DOCX)

S2 Table Plasmodium falciparum HRP2 (Histidine-rich Protein 2) amplification and sequencing (primer sequences). (DOCX)

S1 Dataset (PDF)

## Abbreviations

ACT: Artemisinin-Based Combination Therapy
DBS: Dried Blood Spots
DNA: Deoxyribonucleic Acid
GIS: Geographical Information System
HRP2: Histidine-Rich Protein 2
HRP3: Histidine-Rich Protein 3
IRS: Indoor Residual Spraying
ITNs: Insecticide-Treated Mosquito Nets
LLINs: Long-Lasting Insecticide-Treated Nets
mRDTs: Malaria Rapid Diagnostic Tests
MSP1: Merozoite Surface Antigen 1
MSP2: Merozoite Surface Antigen 2
PCR: Polymerase Chain Reaction
pfhrp2: *Plasmodium falciparum* Histidine-Rich Protein 2
RDTs: Rapid Diagnostic Tests
rRNA: Ribosomal Ribonucleic Acid
WBC: While Blood Cells
WHO: World Health Organization
