# Correction: Limitations of rapid diagnostic tests in malaria surveys in areas with varied transmission intensity in Uganda 2017–2019: Implications for selection and use of HRP2 RDTs

**DOI:** 10.1371/journal.pone.0304728

**Published:** 2024-05-29

**Authors:** Bosco B. Agaba, Joaniter I. Nankabirwa, Adoke Yeka, Sam Nsobya, Karryn Gresty, Karen Anderson, Paul Mbaka, Christiane Prosser, David Smith, Jimmy Opigo, Rhoda Namubiru, Emmanuel Arinaitwe, John Kissa, Samuel Gonahasa, Sungho Won, Bora Lee, Chae Seung Lim, Charles Karamagi, Qin Cheng, Joan K. Nakayaga, Moses R. Kamya

The first author’s name is written incorrectly. The correct name is: Bosco B. Agaba. The correct citation is: Agaba BB, Nankabirwa JI, Yeka A, Nsobya S, Gresty K, Anderson K, et al. (2020) Limitations of rapid diagnostic tests in malaria surveys in areas with varied transmission intensity in Uganda 2017–2019: Implications for selection and use of HRP2 RDTs. PLoS ONE 15(12): e0244457. https://doi.org/10.1371/journal.pone.0244457.
