# Supplementary figures and images for: Limitations of rapid diagnostic tests in malaria surveys in areas with varied transmission intensity in Uganda 2017-2019: Implications for selection and use of HRP2 RDTs
Source: PLoS One. 2020 Dec 31;15(12):e0244457. doi: 10.1371/journal.pone.0244457 (PMC7774953; doi:10.1371/journal.pone.0244457)

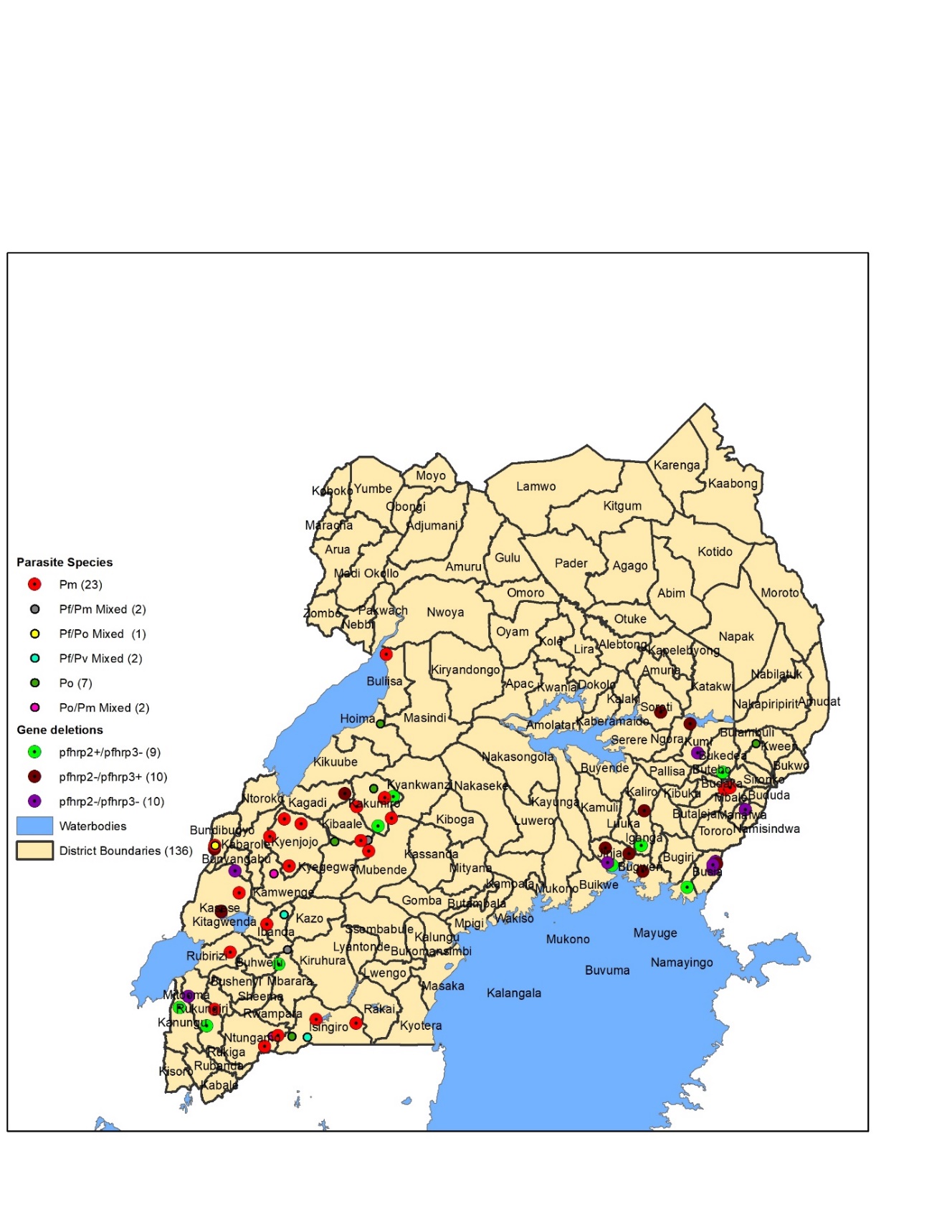


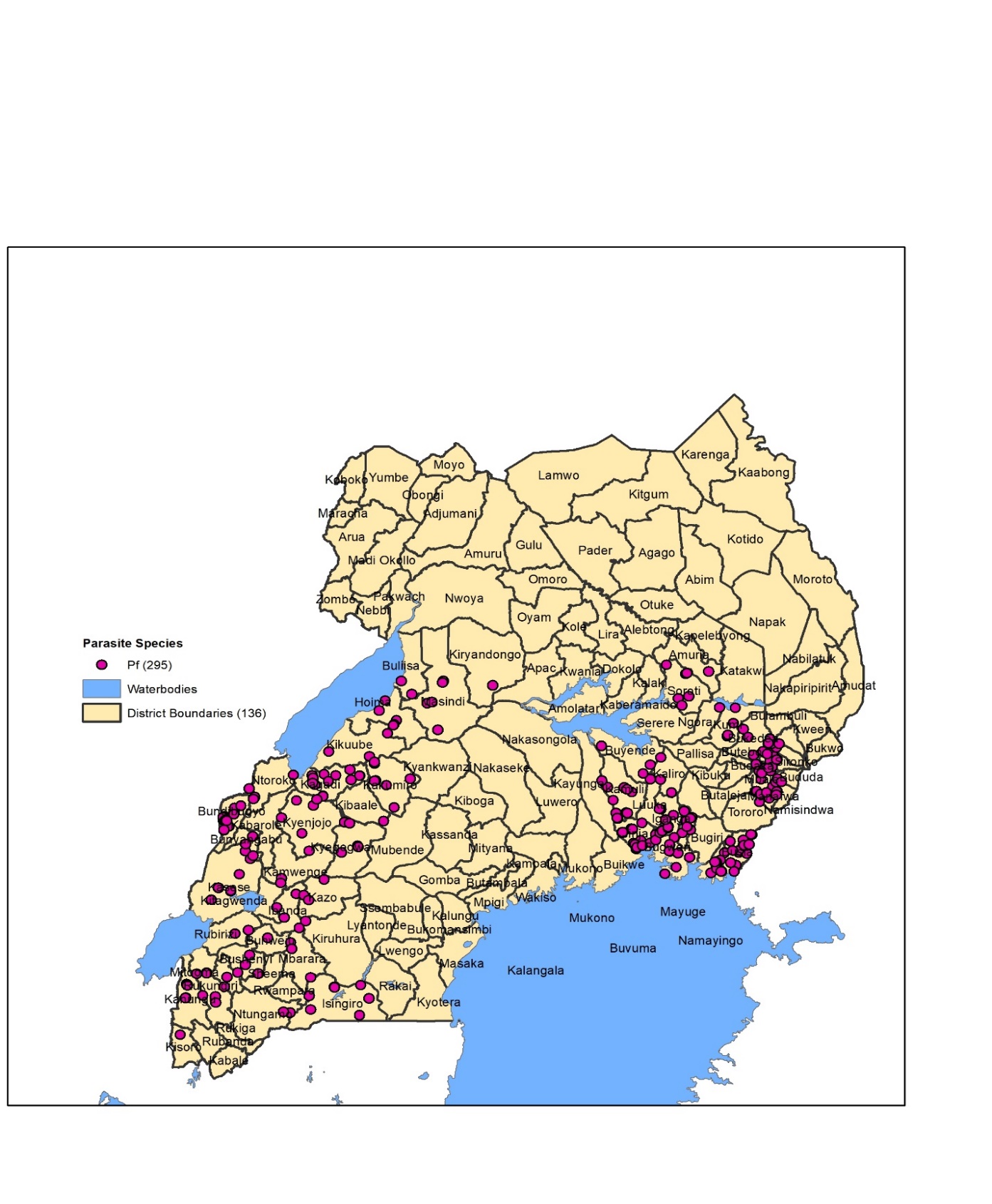

Supplement: S1 Fig — (DOCX) [file pone.0244457.s001.docx]
