# Supplementary material for: Limitations of rapid diagnostic tests in malaria surveys in areas with varied transmission intensity in Uganda 2017-2019: Implications for selection and use of HRP2 RDTs
Source: PLoS One. 2020 Dec 31;15(12):e0244457. doi: 10.1371/journal.pone.0244457 (PMC7774953; doi:10.1371/journal.pone.0244457)
Supplement: S1 Table — (DOCX) [file pone.0244457.s002.docx]

**Supplementary Table 1: Parasite DNA amplification: Primer sequences:**

Single reverse primer and 4 species-specific primers:

| **Rev: GTA TCT GAT CGT CTT CAC TCC C**  **Pf: AAC AGA CGG GTA GTC ATG ATT GAG**  **Pv: CGG CTT GGA AGT CCT TGT**  **Po: CTG TTC TTT GCA TTC CTT ATG C**  **Pm: CGT TAA GAA TAA ACG CCA AGC G** |
| --- |
