# Supplementary material for: Limitations of rapid diagnostic tests in malaria surveys in areas with varied transmission intensity in Uganda 2017-2019: Implications for selection and use of HRP2 RDTs
Source: PLoS One. 2020 Dec 31;15(12):e0244457. doi: 10.1371/journal.pone.0244457 (PMC7774953; doi:10.1371/journal.pone.0244457)
Supplement: S2 Table — (DOCX) [file pone.0244457.s003.docx]

Supplementary Table 2: Plasmodium falciparum HRP2 (Histidine-rich Protein 2) amplification and sequencing (Primer sequences)

| Gene name | Primer sequence |
| --- | --- |
| *Pf hrp2* exon2  *MAL7P1.231*  *Pf3D7_0831800* | F1 5’ CAA AAG GAC TTA ATT TAA ATA AGA G 3’  R1 5’ AAT AAA TTT AAT GGC GTA GGC A 3’  F2 5’ ATT ATT ACA CGA AAC TCA AGC AC 3’ |
| *AMI Pf hrp2* exon 1  *MAL7P1.231*  *Pf3D7_0831800* | F1 5’ tat ccg ctg ccg ttt ttg Cc 3’  RS 5’ AGC ATG ATG GGC ATC ATC TA 3’ |
| *AMI Pfhrp3* exon 1 *MAL13P1.480*  *Pf3D7_1372200* | F 5’ TAT CCG CTG CCG TTT TTG CTT CC 3’  R 5’ TGC ATG ATG GGC ATC ACC TG 3’ |
| *Pfhrp3* exon 2 *MAL13P1.480*  *Pf3D7_1372200* | F1 5’ AAT GCA AAA GGA CTT AAT TC 3’  R1 5’ TGG TGT AAG TGA TGC GTA GT 3’  F2 5’ AAA TAA GAG ATT ATT ACA CGA AAG 3’ |
| *Pfmsp1*  For P.f genotyping | Round 1  O1: CACATGAAAGTTATCAAGAACTTGTC  O2: GTACGTCTAATTCATTTGCACG |
| *Pfmsp1* | Round 2  N1: GCAGTATTGACAGGTTATGG  N2: GATTGAAAGGTATTTGAC |
| *Pfmsp2*  For Pf genotyping | Round 1  S3 : GAAGGTAATTAAAACATTGTC  S2: GAGGGATGTTGCTGCTCCACAG |
| *Pfmsp2* | Round 2  S1: GAGTATAAGGAGAAGTATG  S4: CTAGAACCATGCATATGTCC |
