# Supplementary material for: Limitations of rapid diagnostic tests in malaria surveys in areas with varied transmission intensity in Uganda 2017-2019: Implications for selection and use of HRP2 RDTs
Source: PLoS One. 2020 Dec 31;15(12):e0244457. doi: 10.1371/journal.pone.0244457 (PMC7774953; doi:10.1371/journal.pone.0244457)
Supplement: S1 Dataset — (PDF) [file pone.0244457.s004.pdf]

Region	District	Endemicity (MIS Parasite Rates)	Lat	Lon	Gender	Age	Barcode Label	Survey	RDT	Microscopy	Density	Multiplex PCR	Parasite Species	*pfhrp2-/pfhrp3+*	*pfhrp2+/pfhrp3-*	*pfhrp2-/pfhrp3-*		
Western	Bundibugyo	Low	0.62247	29.99602	Female	6	G678	2	Negative	Positive	48	Postive	Pm					
Western	Bundibugyo	Low	0.62247	29.99602	Male	4	G679	2	Negative	Positive	50	Postive	Pf/Po Mixed					
Western	Bundibugyo	Low	0.72723	30.0716	Female	6	G815	2	Negative	Positive	240	Postive	Pf					
Western	Isingiro	Low	-0.9333	30.62278	Male	10	M267	2	Negative	Positive	620	Postive	Po					
Western	Mitooma	Low	-0.7138	29.99587	Male	5	M426	2	Negative	Positive	272	Postive	Pf					
Western	Mitooma	Low	-0.7132	29.9935	Female	3	M425	2	Negative	Positive	900	Postive	Pm					
Western	Kiryandongo	Low	1.80288	32.09757	Male	4	C220	2	Negative	Positive	48	Postive	Pf					
Western	Kiryandongo	Low	2.21784	32.24252	Female	3	C186	2	Negative	Positive	280	Negative	neg					
Western	Rubirizi	Low	-0.2103	29.89598	Female	9	M866	2	Negative	Positive	240	Negative	neg					
Western	Rubirizi	Low	-0.2726	30.08051	Female	5	M884	2	Negative	Positive	96	Negative	neg					
Western	Ntungamo	Low	-0.9283	30.46065	Female	3	N844	2	Negative	Positive	64	Postive	Po/Pm Mixed					
Western	Ntungamo	Low	1.43189	31.668	Male	4	C347	2	Negative	Positive	700	Postive	Pf					
Western	Masindi	Low	1.8396	31.71062	Male	7	C418	2	Negative	Positive	400	Postive	Pf					
Western	Masindi	Low	1.8229	31.70539	Male	5	C432	2	Negative	Positive	680	Postive	Pf					
Western	Masindi	Low	1.66146	31.72742	Female	3	C587	2	Negative	Positive	16	Negative	neg					
Western	Kasese	Low	0.01103	29.89447	Female	7	J446	2	Negative	Positive	400	Postive	Pf					
Western	Hoima	Low	1.59274	31.20671	Male	5	K161	2	Negative	Positive	520	Postive	Pf					
Western	Hoima	Low	1.67546	31.2552	Female	3	K168	2	Negative	Positive	600	Postive	Pf					
Western	Hoima	Low	1.61789	31.34469	Male	2	K182	2	Negative	Positive	448	Postive	Po					
Western	Bushenyi	Low	-0.4797	30.23892	Male	3	K917	2	Negative	Positive	200	Negative	neg					
Western	Kyegegwa	Low	0.65602	30.96918	Female	3	P081	2	Negative	Positive	800	Postive	Po					
Western	Kyegegwa	Low	0.57756	31.25346	Female	2	P132	2	Negative	Positive	189	Postive	Po					
Western	Kyegegwa	Low	0.57679	31.24993	Female	7	P130	2	Negative	Positive	340	Postive	Pm					
Western	Kyegegwa	Low	0.40798	30.91181	Female	5	P126	2	Negative	Positive	900	Postive	Pf					
Western	Kyenjojo	Low	0.69666	30.44282	Female	5	P708	2	Negative	Positive	760	Postive	Pm					
Western	Kyenjojo	Low	0.80193	30.69989	Male	2	P681	2	Negative	Positive	1400	Postive	Pm					
Western	Kakumiro	Low	1.00833	31.37969	Female	4	Q412	2	Negative	Positive	1317	Postive	Pm					
Western	Kakumiro	Low	1.01329	31.38019	Female	4	Q414	2	Negative	Positive	2167	Postive	Pm					
Western	Kakumiro	Low	0.84734	31.4332	Female	5	Q430	2	Negative	Positive	79	Postive	Po					
Western	Kagadi	Low	0.9874	30.68732	Male	8	Q598	2	Negative	Positive	500	Postive	Pf					
Western	Kagadi	Low	0.9874	30.68732	Female	4	Q599	2	Negative	Positive	32	Postive	Pf					
Western	Kagadi	Low	0.96961	30.70639	Female	3	Q593	2	Negative	Positive	1040	Postive	Pf					
Western	Kagadi	Low	1.04921	30.86949	Female	7	Q560	2	Negative	Positive	80	Postive	Pf					
Western	Kagadi	Low	1.04969	30.68266	Female	7	Q611	2	Negative	Positive	520	Postive	Pf					
Western	Kagadi	Low	0.87966	30.76955	Male	4	Q590	2	Negative	Positive	450	Postive	Pf					
Western	Kagadi	Low	0.87725	30.76946	Female	7	Q588	2	Negative	Positive	420	Postive	Pf					
Western	Kagadi	Low	1.06004	30.77923	Female	4	Q574	2	Negative	Positive	100	Postive	Pf					
Western	Kagadi	Low	1.00098	30.8118	Male	9	Q555	2	Negative	Positive	920	Postive	Pf					
Western	Kagadi	Low	1.0463	31.05806	Male	5	Q761	2	Negative	Positive	680	Postive	Pf					
Western	Kibaale	Low	0.67047	31.24347	Male	2	Q870	2	Negative	Positive	200	Postive	Pf					
Western	Kibaale	Low	0.67088	31.24102	Male	10	Q866	2	Negative	Positive	96	Postive	Pm/Pf Mixed					
Eastern	Namayingo	Moderate	0.24888	33.88823	Male	9	C865	2	Negative	Positive	720	Postive	Pf					
Eastern	Namayingo	Moderate	0.40494	33.91356	Female	6	C882	2	Negative	Positive	2080	Postive	Pf					
Eastern	Namayingo	Moderate	0.32888	33.83743	Female	7	C879	2	Negative	Positive	880	Postive	Pf					
Eastern	Soroti	Low	1.97246	33.45758	Female	10	A459	2	Negative	Positive	32	Postive	Pf					
Eastern	Soroti	Low	1.69263	33.54998	Female	3	A470	2	Negative	Positive	2040	Postive	Pf					
Eastern	Soroti	Low	1.89782	33.6072	Female	2	A501	2	Negative	Positive	96	Postive	Pf					
Eastern	Soroti	Low	1.9022	33.61586	Male	9	A503	2	Negative	Positive	720	Postive	Pf					
Eastern	Soroti	Low	1.71087	33.63081	Female	2	A611	2	Negative	Positive	800	Postive	Pf	1				
Eastern	Jinja	Moderate	0.57553	33.11237	Female	5	D333	2	Negative	Positive	420	Postive	Pf					
Eastern	Kamuli	Moderate	0.73818	33.14342	Female	10	D661	2	Negative	Positive	1600	Postive	Pf					
Eastern	Kamuli	Moderate	0.94905	33.12566	Male	5	D642	2	Negative	Positive	155	Postive	Pf					
Eastern	Busia	Moderate	0.49945	33.95853	Female	6	D925	2	Negative	Positive	300	Postive	Pf					
Eastern	Busia	Moderate	0.42923	34.05726	Male	8	D940	2	Negative	Positive	48	Postive	Pf			1		
Eastern	Busia	Moderate	0.40895	34.02555	Male	3	E068	2	Negative	Positive	32	Postive	Pf					
Eastern	Busia	Moderate	0.49427	34.07887	Female	3	E098	2	Negative	Positive	1680	Postive	Pf			1		
Eastern	Iganga	Moderate	0.62189	33.61959	Female	9	E222	2	Negative	Positive	760	Postive	Pf					
Eastern	Iganga	Moderate	0.59509	33.64493	Female	4	E264	2	Negative	Positive	680	Postive	Pf					
Eastern	Iganga	Moderate	0.56765	33.58038	Male	7	E271	2	Negative	Positive	208	Postive	Pf					
Eastern	Iganga	Moderate	0.61142	33.47232	Female	10	E438	2	Negative	Positive	80	Negative	neg					
Eastern	Iganga	Moderate	0.59163	33.42295	Female	4	E506	2	Negative	Positive	110	Postive	Pf					
Eastern	Luuka	Moderate	1.02032	33.32933	Male	2	E822	2	Negative	Positive	480	Postive	Pf					
Eastern	Ngora	Low	1.61531	33.87298	Male	6	A840	2	Negative	Positive	1120	Postive	Pf	1				
Eastern	Sironko	Low	1.20925	34.29622	Female	9	A905	2	Negative	Positive	560	Postive	Pf					
Eastern	Mayuge	Moderate	0.3729	33.63943	Male	5	F410	2	Negative	Positive	16	Negative	neg					
Eastern	Mayuge	Moderate	0.53103	33.5207	Female	5	F422	2	Negative	Positive	144	Postive	Pf					
Eastern	Mayuge	Moderate	0.22835	33.44653	Female	4	F644	2	Negative	Positive	304	Postive	Pf					
Eastern	Mbale	Low	1.14328	34.15998	Female	9	G024	2	Negative	Positive	1680	Postive	Pf					
Eastern	Mbale	Low	0.91626	34.15684	Male	2	G236	2	Negative	Positive	64	Postive	Pf					
Eastern	Mbale	Low	1.08038	34.15427	Male	2	G312	2	Negative	Positive	2520	Postive	Pm					
Eastern	Manafwa	Low	0.89878	34.32378	Male	2	B547	2	Negative	Positive	1501	Negative	neg					
Eastern	Manafwa	Low	0.85833	34.26184	Female	4	B731	2	Negative	Positive	1400	Postive	Pf					
Eastern	Manafwa	Low	0.88991	34.31717	Female	2	B690	2	Negative	Positive	880	Negative	neg					
Eastern	Manafwa	Low	0.91836	34.29992	Male	5	B666	2	Negative	Positive	600	Postive	Pf					
Eastern	Buyende	Moderate	1.1376	33.33199	Female	4	G560	2	Negative	Positive	3840	Postive	Pf					
Western	Kyegegwa	Low	1.80158	32.09829	Male	10	C217	2	Negative	Positive	860	Postive	Pf					
Western	Kyegegwa	Low	0.31296	30.0839	Female	6	J755	1	Negative	Positive	640	Negative	neg					
Eastern	Kanungu	Low	0.99022	34.32096	Male	6	B705	1	Negative	Positive	3280	Postive	Pf					
Western	Kisoro	Low	1.20718	31.13083	Male	7	K314	2	Negative	Positive	2560	Postive	Pf					
Western	Rukungiri	Low	1.04921	30.86949	Female	7	Q561	2	Negative	Positive	6640	Postive	Pf					
Eastern	Iganga	Low	0.39884	34.08573	Male	9	E095	2	Negative	Positive	400	Postive	Pf					
Eastern	Namayingo	Low	1.11116	34.23027	Female	2	G077	2	Negative	Positive	6560	Postive	Pf					
Eastern	Bukedea	Low	1.23831	34.23294	Female	6	B058	2	Negative	Positive	288	Postive	Pf					
Western	Bukedea	Low	1.04725	31.05393	Male	5	Q765	2	Negative	Positive	7440	Postive	Pf	1				
Eastern	Bulambuli	Low	1.28613	34.31809	Female	2	B217	1	Negative	Positive	16	Postive	Pf					
Eastern	Bukedea	Low	1.25093	34.21516	Male	4	B426	1	Negative	Positive	760	Postive	Pf					
Eastern	Bulambuli	Low	1.31678	34.27852	Female	7	B265	1	Negative	Positive	112	Negative	neg					
Eastern	Bukedea	Low	1.3692	34.09668	Male	4	B431	1	Negative	Positive	800	Postive	Pf					

Eastern	Kumi	Low	1.37887	33.93436	Female	4	A021	1	Negative	Positive	4240	Postive	Pf			1		
Eastern	Bukedea	Low	1.3692	34.09668	Male	10	B429	1	Negative	Positive	680	Negative	neg					
Eastern	Kumi	Low	1.61451	33.99704	Female	6	A052	1	Negative	Positive	3532	Postive	Pf					
Eastern	Bulambuli	Low	1.31714	34.25734	Male	2	B203	1	Negative	Positive	500	Postive	Pf					
Eastern	Manafwa	Low	0.82687	34.18806	Male	4	B737	1	Negative	Positive	1400	Postive	pf					
Eastern	Bukedea	Low	1.25093	34.21516	Male	5	B425	1	Negative	Positive	1200	Postive	Pf					
Eastern	Kumi	Low	1.48615	33.98262	Female	6	A024	1	Negative	Positive	1106	Postive	Pf					
Eastern	Bulambuli	Low	1.28613	34.31809	Male	5	B216	1	Negative	Positive	112	Negative	neg					
Eastern	Bukedea	Low	1.43814	34.0597	Female	4	B462	1	Negative	Positive	64	Postive	Pf					
Eastern	Bulambuli	Low	1.31678	34.27852	Female	9	B263	1	Negative	Positive	1600	Negative	neg					
Eastern	Bukedea	Low	1.22207	34.13763	Female	4	B384	1	Negative	Positive	32	Postive	Pf		1			
Eastern	Manafwa	Low	0.91787	34.29987	Female	2	B683	1	Negative	Positive	64	Negative	neg					
Eastern	Manafwa	Low	0.91475	34.32232	Female	5	B515	1	Negative	Positive	64	Postive	Pf			1		
Eastern	Bulambuli	Low	1.31797	34.25293	Female	6	B209	1	Negative	Positive	560	Postive	Pf					
Eastern	Bukedea	Low	1.36963	34.09644	Male	8	B453	1	Negative	Positive	320	Negative	neg					
Eastern	Bulambuli	Low	1.35657	34.2694	Female	6	B284	1	Negative	Positive	80	Postive	Pf					
Eastern	Namayingo	Moderate	0.40577	33.91283	Female	5	C930	1	Negative	Positive	32	Postive	Pf					
Eastern	Mbale	Low	1.11102	34.25361	Male	5	G003	1	Negative	Positive	1840	Negative	neg					
Eastern	Kamuli	Moderate	0.95385	32.99947	Female	2	D535	1	Negative	Positive	112	Postive	Pf					
Eastern	Mayuge	Moderate	0.511	33.37368	Male	9	F257	1	Negative	Positive	400	Postive	Pf					
Eastern	Namayingo	Moderate	0.27942	33.85201	Female	3	C933	1	Negative	Positive	320	Postive	Pf					
Eastern	Jinja	Moderate	0.50873	33.25649	Male	5	D073	1	Negative	Positive	900	Postive	Pf					
Eastern	Iganga	Moderate	0.76665	33.4087	Male	7	E560	1	Negative	Positive	32	Postive	Pf					
Eastern	Namayingo	Moderate	0.27942	33.85201	Male	5	C932	1	Negative	Positive	2840	Postive	Pf					
Eastern	Mayuge	Low	0.41314	33.48692	Female	4	F302	1	Negative	Positive	286	Postive	Pf	1				
Eastern	Jinja	Moderate	0.46284	33.2378	Male	7	D033	1	Negative	Positive	2160	Postive	Pf		1			
Eastern	Mayuge	Low	0.50972	33.37376	Female	4	F254	1	Negative	Positive	320	Postive	Pf					
Eastern	Mayuge	Low	0.55639	33.37249	Female	2	F269	1	Negative	Positive	480	Postive	Pf	1				
Western	Ntoroko	Low	0.85929	30.22504	Female	3	C725	1	Negative	Positive	96	Postive	Pf					
Eastern	Busia	Low	0.46333	34.06269	Male	10	D918	1	Negative	Positive	250	Postive	Pf			1		
Eastern	Mbale	Low	1.08749	34.18474	Male	2	G331	1	Negative	Positive	48	Postive	Pf					
Eastern	Jinja	Moderate	0.48354	33.20101	Male	4	D096	1	Negative	Positive	48	Postive	Pf			1		
Eastern	Namayingo	Moderate	0.28267	33.84817	Female	5	C934	1	Negative	Positive	128	Postive	Pf		1			
Eastern	Jinja	Moderate	0.60345	33.18084	Male	9	D343	1	Negative	Positive	400	Postive	Pf	1				
Eastern	Jinja	Moderate	0.60596	33.18176	Female	7	D347	1	Negative	Positive	320	Postive	Pf					
Eastern	Jinja	Moderate	0.47597	33.21293	Male	3	D100	1	Negative	Positive	96	Postive	Pf					
Eastern	Iganga	Moderate	0.75151	33.59687	Male	2	E300	1	Negative	Positive	520	Postive	Pf					
Eastern	Busia	Low	0.30121	34.0031	Male	9	D963	1	Negative		63	Postive	Pf					
Eastern	Mbale	Low	0.91936	34.16602	Male	2	G198	1	Negative	Positive	720	Postive	Pf					
Eastern	Mbale	Moderate	0.89374	34.19586	Male	4	G217	1	Negative	Positive	950	Postive	Pf					
Eastern	Namayingo	Moderate	0.23738	33.80531	Male	2	C888	1	Negative	Positive	400	Postive	Pf					
Eastern	Mbale	Low	1.09481	34.20027	Female	9	G053	1	Negative	Positive	600	Postive	Pm					
Eastern	Namayingo	Moderate	0.34364	33.87968	Male	2	C869	1	Negative	Positive	125	Postive	Pf					
Eastern	Iganga	Moderate	0.58717	33.42425	Male	3	E507	1	Negative	Positive	640	Postive	Pf					
Eastern	Busia	Low	0.47417	34.08865	Male	5	D835	1	Negative	Positive	520	Postive	Pf	1				
Eastern	Mbale	Low	1.08559	34.27245	Male	5	G028	1	Negative	Positive	100	Postive	Pf					
Eastern	Busia	Moderate	0.42367	33.9775	Female	6	E053	1	Negative	Positive	50	Postive	Pf					
Eastern	Mayuge	Moderate	0.48644	33.33036	Female	5	F323	1	Negative	Positive	150	Postive	Pf					
Eastern	Kaliro	Moderate	1.19878	33.41158	Female	2	F721	1	Negative	Positive	400	Postive	Pf					
Eastern	Namayingo	Moderate	0.32731	33.83952	Male	9	C912	1	Negative	Positive	980	Postive	Pf					
Eastern	Kamuli	Moderate	0.68859	33.07469	Male	5	D743	1	Negative	Positive	3400	Postive	Pf					
Eastern	Busia	Moderate	0.46641	34.05926	Female	4	D919	1	Negative	Positive	50	Postive	pf			1		
Eastern	Mayuge	Moderate	0.45561	33.48529	Female	5	F307	1	Negative	Positive	600	Negative	neg					
Eastern	Jinja	Moderate	0.60978	33.12351	Male	3	D333	1	Negative	Positive	50	Negative	neg					
Eastern	Busia	Moderate	0.46794	34.06332	Female	3	D917	1	Negative	Positive	31	Postive	Pf					
Eastern	Mayuge	Moderate	0.47329	33.4829	Male	2	F447	1	Negative	Positive	160	Negative	neg					
Eastern	Jinja	Moderate	0.57408	33.1133	Male	5	D340	1	Negative	Positive	150	Postive	Pf					
Eastern	Mayuge	Moderate	0.51073	33.37363	Male	10	F255	1	Negative	Positive	760	Postive	pf					
Eastern	Kaliro	Moderate	0.90702	33.49839	Female	10	F715	1	Negative	Positive	560	Postive	pf	1				
Eastern	Jinja	Moderate	0.60168	33.1942	Female	9	D351	1	Negative	Positive	223	Postive	pf					
Eastern	Iganga	Moderate	0.62019	33.61872	Female	5	E220	1	Negative	Positive	48	Postive	Pf					
Eastern	Mayuge	Moderate	0.40154	33.54643	Male	5	F497	1	Negative	Positive	420	Postive	Pf					
Eastern	Busia	Moderate	0.42215	34.02469	Female	3	E113	1	Negative	Positive	112	Postive	pf					
Eastern	Busia	Moderate	0.5108	34.06016	Female	8	E140	1	Negative	Positive	640	Postive	Pf					
Eastern	Mayuge	Moderate	0.36453	33.63709	Male	2	F412	1	Negative	Positive	380	Postive	Pf					
Western	Masindi	Low	1.65276	31.58428	Male	6	C261	1	Negative	Positive	520	Postive	Pf					
Eastern	Namayingo	Moderate	0.3263	33.84753	Male	5	C914	1	Negative	Positive	80	Postive	Pf					
Eastern	Jinja	Moderate	0.44954	33.23029	Female	4	D229	1	Negative	Positive	64	Postive	Pf					
Western	Bundibugyo	Moderate	0.78007	30.07848	Female	4	G621	1	Negative	Positive	144	Postive	pf					
Western	Kyenjojo	Low	0.84009	30.56264	Male	10	P674	1	Negative	Positive	2120	Postive	Pm					
Western	Kamuli	Moderate	0.41625	30.16142	Female	5	J924	1	Negative	Positive	720	Postive	Pf			1		
Western	Kasese	Low	0.35403	30.19929	Male	3	J980	1	Negative	Positive	80	Postive	Pf					
Western	Isingiro	Low	-0.6381	30.67122	Male	2	M111	1	Negative	Positive	16	Postive	Pf					
Western	Isingiro	Low	-0.7166	30.85848	Male	3	M193	1	Negative	Positive	1920	Postive	Pf					
Western	Kyegegwa	Low	0.4631	31.03877	Female	6	P085	1	Negative	Positive	50	Postive	pf					
Western	Kasese	Low	0.08276	30.04678	Female	4	J832	1	Negative	Positive	960	Postive	pf			1		
Western	Bundibugyo	Low	0.67692	29.98965	Male	4	G855	1	Negative	Positive	680	Postive	pf					
Western	Kamwengye	Low	-0.0514	30.40781	Female	2	N194	1	Negative	Positive	100	Postive	Pf					
Western	Kasese	Low	0.11183	29.95067	Female	3	J614	1	Negative	Positive	880	Postive	Pf					
Western	Kabaale	Low	1.01364	30.98932	Male	2	Q805	1	Negative	Positive	240	Postive	pf					
Western	Kanungu	Low	-0.8069	29.69094	Female	10	P977	1	Negative	Positive	400	Postive	Pf					
Western	Ibanda	Low	0.06004	30.55758	Female	4	J064	1	Negative	Positive	32	Postive	Pf					
Western	Rubirizi	Low	-0.2473	30.12174	Female	4	M884	1	Negative	Positive	1280	Postive	pm					
Western	Kasese	Low	0.23601	30.19266	Male	2	J729	1	Negative	Positive	620	Postive	Pm					
Western	Kibaale	Low	0.96858	31.265	Female	4	Q280	1	Negative	Positive	224	Postive	Pf					
Western	Bundibugyo	Low	0.59258	29.99309	Female	6	G727	1	Negative	Positive	112	Postive	pf					
Western	Ibanda	Low	0.06073	30.5577	Female	8	J058	1	Negative	Positive	380	Postive	Pf/Pv Mixed					
Western	Kyenjojo	Low	0.80028	30.69302	Male	2	P665	1	Negative	Positive	1040	Postive	Pf					
Western	Kibaale	Low	0.84723	31.43683	Male	3	Q415	1	Negative	Positive	390	Postive	Pm					
Western	Isingiro	Low	-0.8263	31.14649	Male	3	L969	1	Negative	Positive	330	Postive	Pm					

Western	Bundibugyo	Low	0.72849	30.07015	Male	10	G834	1	Negative	Positive	96	Postive	Pf					
Western	Isingiro	Low	-0.9391	30.74714	Male	2	M262	1	Negative	Positive	440	Postive	Pf/Pv Mixed					
Western	Kibaale	Low	0.94055	31.15371	Male	8	Q304	1	Negative	Positive	3360	Postive	Pm					
Western	Kibaale	Low	0.79454	31.30346	Male	4	Q267	1	Negative	Positive	920	Negative	neg					
Western	Ibanda	Low	-0.019	30.42023	Female	7	J080	1	Negative	Positive	1360	Postive	Pm					
Western	Kyenjojo	Low	0.80028	30.69302	Female	6	P664	1	Negative	Positive	50	Postive	Pf					
Western	Kamwengye	Low	0.19126	30.44243	Male	2	N287	1	Negative	Positive	16	Postive	Pf					
Western	Kiruhura	Low	0.05071	30.62056	Male	8	J122	1	Negative	Positive	80	Postive	Pf					
Western	Ntungamo	Low	-0.9275	30.51144	Female	3	N860	1	Negative	Positive	1920	Postive	Pm					
Western	Bundibugyo	Low	0.7958	30.12497	Female	9	G688	1	Negative	Positive	220	Negative	neg					
Western	Isingiro	Low	-0.7953	30.81986	Male	7	M133	1	Negative	Positive	1920	Postive	Pm					
Western	Buhweju	Low	-0.3065	30.33511	Male	2	R030	1	Negative	Positive	160	Postive	Pf					
Western	Busia	Moderate	-0.6062	29.77975	Male	5	P358	1	Negative	Positive	620	Postive	Pf			1		
Western	Bundibugyo	Low	0.70997	29.99645	Female	9	G768	1	Negative	Positive	32	Postive	Pf					
Western	Kiruhura	Low	-0.1627	30.63112	Male	5	J307	1	Negative	Positive	96	Negative	neg					
Western	Kibaale	Low	0.66325	31.18703	Male	10	Q875	1	Negative	Positive	112	Postive	Pm					
Western	Ntungamo	Low	-0.9304	30.51215	Male	9	N862	1	Negative	Positive	400	Postive	Pf					
Western	Ntungamo	Low	-1.0148	30.39931	Male	7	N782	1	Negative	Positive	640	Postive	Pm					
Western	Iganga	Moderate	0.78334	31.3256	Male	2	Q253	1	Negative	Positive	400	Postive	Pf		1			
Western	Rukungiri	Low	-0.7866	29.82745	Male	7	P374	1	Negative	Positive	288	Postive	Pf					
Western	Ibanda	Low	-0.2268	30.58531	Male	6	J026	1	Negative	Positive	1400	Postive	Pf/Pm Mixed					
Western	Ibanda	Low	0.71424	30.35763	Female	10	L244	1	Negative	Positive	336	Negative	neg					
Western	Rukungiri	Low	-0.5976	29.85857	Female	3	P368	1	Negative	Positive	660	Postive	Pf					
Western	Bundibugyo	Low	0.72753	30.0717	Female	7	G845	1	Negative	Positive	520	Postive	Pf					
Western	Kamwengye	Low	0.3963	30.47288	Female	3	N243	1	Negative	Positive	460	Postive	Pm/Po Mixed					
Western	Kasese	Low	0.08882	30.04734	Female	10	J821	1	Negative	Positive	144	Postive	Pf	1				
Western	Kyenjojo	Low	0.45518	30.60132	Female	10	P814	1	Negative	Positive	820	Postive	Pm					
Western	Kibaale	Low	1.15064	31.17137	Female	6	Q319	1	Negative	Positive	1120	Postive	Pf					
Western	Kyenjojo	Low	0.45518	30.60132	Female	4	P816	1	Negative	Positive	1680	Postive	Pm					
Western	Kiruhura	Low	0.00901	30.65546	Male	4	J132	1	Negative	Positive	720	Postive	Pf					
Western	Rukungiri	Low	-0.7956	29.92402	Female	7	P510	1	Negative	Positive	180	Postive	Pf					
Eastern	Busia	Moderate	1.31714	34.25734	Female	2	B204	1	Negative	Positive	32	Postive	Pf					
Eastern	Busia	Moderate	1.31642	34.27808	Female	4	B262	1	Negative	Positive	144	Postive	Pf					
Eastern	Busia	Moderate	1.43814	34.0597	Male	6	B461	1	Negative	Positive	208	Postive	Pf					
Western	Kasese	Low	-0.7166	30.85848	Male	7	M195	1	Negative	Positive	880	Postive	Pf					
Western	Kasese	Low	2.18239	31.39256	Female	10	C083	1	Negative	Positive	120	Postive	Pm					
Western	Kisoro	Low	-0.5942	30.10345	Female	10	K811	1	Negative	Positive	144	Postive	Pf					
Western	Mayuge	Moderate	-0.3446	30.51731	Female	6	L583	1	Negative	Positive	4480	Postive	Pf		1			
Eastern	Mbale	Low	0.73642	33.06217	Male	6	D732	1	Negative	Positive	1120	Postive	Pf					
Eastern	Mbale	Low	0.56952	33.11245	Female	5	D342	1	Negative	Positive	1251	Postive	Pf					
Eastern	Bugiri	Moderate	0.46794	34.06332	Female	5	D915	1	Negative	Positive	520	Postive	Pf					
Eastern	Luuka	Moderate	0.5695	33.40768	Male	2	E883	1	Positive	Positive	520	Postive	Pf					
Eastern	Iganga	Moderate	0.68594	33.44975	Female	6	E678	1	Positive	Positive	800	Negative	neg					
Eastern	Iganga	Moderate	0.71922	33.51451	Male	4	E697	1	Positive	Positive	8560	Postive	Pf					
Eastern	Iganga	Moderate	0.76666	33.41236	Male	3	E574	1	Positive	Positive	41960	Postive	Pf					
Eastern	Iganga	Moderate	0.58717	33.42425	Male	2	E508	1	Positive	Positive	4040	Postive	Pf					
Eastern	Iganga	Moderate	0.61087	33.47172	Female	10	E418	1	Positive	Positive	112	Postive	Pf					
Eastern	Iganga	Moderate	0.62231	33.47475	Male	6	E367	1	Positive	Positive	12000	Postive	Pf		1			
Eastern	Iganga	Moderate	0.70025	33.65549	Male	2	E236	1	Positive	Positive	32160	Postive	Pf					
Eastern	Iganga	Moderate	0.66977	33.61203	Male	6	E216	1	Positive	Positive	144	Postive	Pf					
Eastern	Buyende	Moderate	1.3994	32.87886	Male	8	G490	1	Positive	Positive	6480	Negative	neg					
Eastern	Buyende	Moderate	1.29636	32.95131	Male	5	G541	1	Positive	Positive	440	Postive	Pf					
Eastern	Kamuli	Moderate	0.8438	33.04163	Male	7	D738	1	Positive	Positive	2280	Postive	Pf					
Eastern	Kamuli	Moderate	0.73537	33.15161	Male	4	D653	1	Positive	Positive	2320	Postive	Pf					
Eastern	Kamuli	Moderate	0.90963	33.18792	Female	4	D487	1	Positive	Positive	3587	Postive	Pf					
Eastern	Kamuli	Moderate	1.00761	32.95409	Male	6	D526	1	Positive	Positive	2691	Postive	Pf					
Eastern	Jinja	Moderate	0.65546	33.26528	Male	3	D371	1	Positive	Positive	16477	Postive	Pf					
Eastern	Jinja	Moderate	0.57578	33.1126	Female	5	D337	1	Positive	Positive	3670	Postive	Pf					
Eastern	Jinja	Moderate	0.43452	33.22061	Male	5	D224	1	Positive	Positive	1840	Postive	Pf					
Eastern	Jinja	Moderate	0.44962	33.23126	Male	6	D236	1	Positive	Positive	13200	Postive	Pf					
Western	Kibaale	Low	1.00718	31.18097	Male	7	Q325	1	Positive	Positive	5600	Postive	Pf					
Western	Kibaale	Low	1.02944	30.68962	Female	2	Q676	1	Positive	Positive	480	Postive	Pf					
Western	Kibaale	Low	0.98518	30.68682	Female	7	Q562	1	Positive	Positive	4980	Postive	Pf					
Western	Kibaale	Low	1.09107	31.2908	Male	6	Q291	1	Positive	Positive	1160	Postive	Po					
Eastern	Bulambuli	Low	1.45505	34.40991	Female	7	B222	1	Positive	Positive	4600	Postive	Po					
Eastern	Bulambuli	Low	1.35601	34.26838	Female	2	B281	1	Positive	Positive	33600	Postive	Pf					
Eastern	Mbale	Low	1.11081	34.25349	Male	5	G010	1	Positive	Positive	32	Postive	Pf					
Eastern	Mbale	Low	1.11054	34.23028	Female	7	G026	1	Positive	Positive	880	Postive	Pf					
Eastern	Busia	Moderate	0.47449	34.10467	Male	3	D758	1	Positive	Positive	84000	Postive	Pf					
Eastern	Busia	Moderate	0.55447	34.12655	Male	6	D936	1	Positive	Positive	6000	Postive	Pf					
Eastern	Busia	Moderate	0.55601	34.11851	Female	9	D944	1	Positive	Positive	317	Postive	Pf					
Eastern	Busia	Moderate	0.24851	33.989	Female	3	E119	1	Positive	Positive	4240	Postive	Pf					
Eastern	Busia	Moderate	0.4241	33.97776	Male	7	E056	1	Positive	Positive	2480	Postive	Pf					
Eastern	Manafwa	Low	0.95901	34.29031	Male	3	B526	1	Positive	Positive	40258	Postive	Pf					
Eastern	Manafwa	Low	0.91703	34.32143	Male	3	B510	1	Positive	Positive	20960	Postive	pf					
Eastern	Manafwa	Low	0.99316	34.31573	Female	7	B698	1	Positive	Positive	1840	Postive	pf					
Eastern	Manafwa	Low	0.88865	34.31733	Male	2	B752	1	Positive	Positive	4800	Postive	pf					
Eastern	Manafwa	Low	1.02342	34.32527	Female	9	B890	1	Positive	Positive	520	Postive	pf					
Eastern	Bududa	Low	0.99455	34.35652	Female	6	B812	1	Positive	Positive	16000	Postive	Pf					
Eastern	Sironko	Low	1.18094	34.32071	Female	7	A901	1	Positive	Positive	2480	Postive	Pf					
Eastern	Sironko	Low	1.18018	34.32337	Female	3	A905	1	Positive	Positive	16000	Postive	Pf					
Eastern	Sironko	Low	1.16764	34.26906	Male	4	B116	1	Positive	Positive	3680	Postive	Pf					
Eastern	Sironko	Low	1.12154	34.2832	Male	8	B073	1	Positive	Positive	520	Postive	Pf					
Eastern	Kapchorwa	Low	1.31668	34.33881	Male	2	E983	1	Positive	Positive	128	Postive	Pf					
Western	Mitooma	Low	-0.6329	30.01562	Female	2	M441	1	Positive	Positive	920	Postive	Pf					
Western	Mitooma	Low	-0.7137	29.99585	Female	4	M470	1	Positive	Positive	4360	Postive	Pf					
Western	Hoima	Low	1.50998	31.34571	Male	4	K230	1	Positive	Positive	2000	Postive	Pf					
Western	Hoima	Low	1.50998	31.34571	Male	7	K229	1	Positive	Positive	9480	Postive	Pf					
Western	Hoima	Low	1.47594	31.32535	Female	4	K480	1	Positive	Positive	7680	Postive	Pf					
Western	Hoima	Low	1.40173	31.2747	Male	7	K500	1	Positive	Positive	80	Postive	Pf					

Western	Kyegegwa	Low	0.66131	30.93671	Male	2	P190	1	Positive	Positive	47560	Postive	Pf					
Western	Kyegegwa	Low	0.45352	31.0458	Female	2	P173	1	Positive	Positive	1920	Postive	Pf					
Western	Kabarole	Low	0.47816	30.16346	Male	2	L083	1	Positive	Positive	5040	Postive	Pf					
Western	Kabarole	Low	0.38093	30.22084	Female	5	L094	1	Positive	Positive	32	Postive	Pf					
Western	Ntoroko	Low	0.87016	30.23189	Female	8	C722	1	Positive	Positive	41780	Postive	Pf					
Western	Kyenjojo	Low	0.8501	30.72111	Female	4	P699	1	Positive	Positive	65669	Postive	Pf					
Western	Kyenjojo	Low	0.84009	30.56264	Male	7	P675	1	Positive	Positive	8080	Postive	Pf					
Western	Kyenjojo	Low	0.56457	30.60386	Male	4	P895	1	Positive	Positive	26400	Postive	Pf					
Western	Kyenjojo	Low	0.41972	30.65842	Female	3	P850	1	Positive	Positive	88000	Postive	Pf					
Western	Bundibugyo	Low	0.79703	30.12714	Female	4	G695	1	Positive	Positive	2720	Postive	Pf					
Western	Bundibugyo	Low	0.62527	30.0347	Male	10	G644	1	Positive	Positive	520	Postive	Pf					
Western	Buliisa	Low	1.83845	31.38195	Female	10	B977	1	Positive	Positive	5000	Postive	Pf					
Western	Bundibugyo	Low	0.69973	29.9853	Female	2	G776	1	Positive	Positive	48440	Postive	Pf					
Western	Buliisa	Low	1.73044	31.46258	Male	4	B967	1	Positive	Positive	70000	Postive	Pf					
Western	Isingiro	Low	-0.8099	31.12836	Male	5	M045	1	Positive	Positive	82920	Postive	Pf					
Western	Isingiro	Low	-0.9044	30.67262	Male	4	M378	1	Positive	Positive	96	Postive	Pf					
Western	Isingiro	Low	-0.7182	30.85977	Male	6	M192	1	Positive	Positive	5120	Postive	Pf					
Western	Isingiro	Low	-0.7895	30.66052	Male	5	M180	1	Positive	Positive	8000	Postive	Pf					
Western	Isingiro	Low	-0.9517	31.05489	Female	7	L978	1	Positive	Positive	48	Postive	Pf					
Western	Ibanda	Low	-0.2244	30.58273	Male	5	J023	1	Positive	Positive	31360	Postive	Pf					
Western	Ibanda	Low	-0.1369	30.46338	Male	5	J055	1	Positive	Positive	2080	Postive	Pf					
Western	Bushenyi	Low	-0.6069	30.26324	Female	2	K938	1	Positive	Positive	2720	Postive	Pf					
Western	Bushenyi	Low	-0.4487	30.19371	Female	7	K770	1	Positive	Positive	18760	Postive	Pf					
Western	Mbarara	Low	-0.3979	30.52647	Female	10	L545	1	Positive	Positive	520	Postive	Pf					
Western	Kanungu	Low	-0.6927	29.70103	Female	4	Q118	1	Positive	Positive	22240	Postive	Pf					
Western	Kanungu	Low	-0.8038	29.69265	Female	10	P983	1	Positive	Positive	76560	Postive	Pf					
Western	Kanungu	Low	-0.6977	29.70518	Male	6	Q113	1	Positive	Positive	71160	Postive	Pf		1			
Western	Bushenyi	Low	-0.5312	30.15977	Male	5	K631	1	Positive	Positive	5500	Postive	Pf					
Western	Kanungu	Low	-0.8038	29.69265	Female	5	P984	1	Positive	Positive	27840	Postive	Pf					
Western	Bushenyi	Low	-0.6034	30.26017	Male	2	K935	1	Positive	Positive	760	Postive	Pf					
Western	Rukungiri	Low	-0.5994	29.85703	Male	5	P361	1	Positive	Positive	65360	Postive	Pf					
Western	Rukungiri	Low	-0.5994	29.85703	Male	7	P360	1	Positive	Positive	62200	Postive	Pf					
Western	Iganga	Moderate	-0.8484	29.92874	Male	5	P206	1	Positive	Positive	320	Postive	Pf		1			
Eastern	Namayingo	Low	0.26896	33.85029	Female	10	C854	1	Positive	Positive	8640	Postive	Pf					
Western	Bundibugyo	Low	0.67438	30.00245	Male	6	G857	1	Positive	Positive	43892	Postive	Pf					
Western	Ntoroko	Low	0.94067	30.19045	Male	5	C770	1	Positive	Positive	10600	Postive	Pf					
Eastern	Iganga	Moderate	0.77098	33.4003	Male	4	E525	1	Positive	Positive	1936	Postive	Pf					
Northern	Kumi	Low	1.38831	33.94136	Female	7	A006	2	Positive	Positive	3834	Postive	Pf					
Eastern	Iganga	Moderate	0.61738	33.47086	Female	8	E353	2	Positive	Positive	1280	Postive	Pf					
Eastern	Iganga	Moderate	0.6823	33.4515	Female	5	E704	2	Positive	Positive	4320	Postive	Pf					
Western	Ntoroko	Low	0.85308	30.22368	Female	10	C764	2	Positive	Positive	80	Postive	pf					
Eastern	Bulambuli	Low	1.31968	34.25753	Female	7	B204	2	Positive	Positive	19320	Postive	Pf					
Eastern	Mbale	Low	1.01961	34.19998	Female	10	G172	2	Positive	Positive	1056	Postive	Pf					
Western	Kakumiro	Low	1.16302	31.17177	Female	5	Q267	2	Positive	Positive	2380	Postive	Pf					
Western	Rubirizi	Low	-0.2715	30.08116	Male	7	M882	2	Positive	Positive	8040	Postive	Pf					
Western	Rubirizi	Low	-0.2399	30.18295	Male	2	N064	2	Positive	Positive	4720	Postive	Pf					
Eastern	Busia	Moderate	0.3217	33.96199	Female	8	D912	2	Positive	Positive	6840	Postive	Pf					
Eastern	Namayingo	Moderate	0.3281	33.83659	Male	4	C875	2	Positive	Positive	3680	Postive	Pf					
Eastern	Mayuge	Moderate	0.28396	33.53331	Male	3	F591	2	Positive	Positive	680	Postive	Pf					
Western	Kanungu	Low	-0.8072	29.69177	Male	7	Q073	2	Positive	Positive	480	Postive	Pf					
Western	Isingiro	Low	-0.7007	31.0635	Male	7	L990	2	Positive	Positive	3080	Postive	Pf					
Western	Bundibugyo	Low	0.59635	29.99236	Female	4	G603	2	Positive	Positive	5184	Postive	Pf					
Western	Kabarole	Low	0.54197	30.2173	Female	10	L065	2	Positive	Positive	1386	Postive	Pf					
Eastern	Buyende	Low	1.06471	33.27607	Female	7	G541	2	Positive	Positive	346	Postive	Pf					
Eastern	Kabarole	Moderate	0.4504	33.22792	Male	7	D165	2	Positive	Positive	2080	Postive	Pf					
Western	Kasese	Low	0.22511	30.11574	Male	5	K029	2	Positive	Positive	32	Postive	pf					
Western	Kyegegwa	Low	0.65171	30.97767	Male	9	P075	2	Positive	Positive	4000	Postive	Pf					
Eastern	Manafwa	Low	0.85175	34.32683	Female	2	B503	2	Positive	Positive	334	Negative	neg					
Eastern	Kamuli	Moderate	0.9334	33.14948	Male	6	D673	2	Positive	Positive	4350	Postive	Pf					
Eastern	Jinja	Moderate	0.57549	33.11371	Male	6	D339	2	Positive	Positive	57320	Postive	Pf					
Eastern	Manafwa	Low	0.90563	34.24259	Male	5	B725	2	Positive	Positive	800	Postive	Pf					
Western	Kiruhura	Low	-0.1642	30.63282	Female	2	J337	2	Positive	Positive	176	Postive	Pf					
Eastern	Kamuli	Moderate	0.69444	33.07265	Male	10	D620	2	Positive	Positive	638	Postive	Pf					
Western	Kanungu	Low	-0.7044	29.70521	Female	4	Q102	2	Positive	Positive	2640	Postive	pf					
Western	Masindi	Low	1.6637	31.62317	Male	7	C567	2	Positive	Positive	11200	Postive	Pf					
Northern	Amuria	Low	1.91919	33.78849	Male	2	A181	2	Positive	Positive	3920	Postive	Pf					
Eastern	Sironko	Low	1.22912	34.24738	Female	3	B098	2	Positive	Positive	985	Postive	Pf					
Western	Bundibugyo	Low	0.59635	29.99236	Male	7	G602	2	Positive	Positive	8720	Postive	Pf	1				
Western	Kakumiro	Low	1.0044	31.17001	Male	10	Q327	2	Positive	Positive	400	Postive	Pf					
Western	Kagadi	Low	1.09884	30.97836	Female	6	Q772	2	Positive	Positive	2240	Postive	pf					
Eastern	Jinja	Moderate	0.46506	33.26938	Male	3	D118	2	Positive	Positive	5320	Postive	Pf					
Eastern	Sironko	Low	1.18108	34.31945	Female	8	A917	2	Positive	Positive	2800	Postive	Pf					
Eastern	Iganga	Moderate	0.69097	33.64584	Male	8	E229	2	Positive	Positive	1360	Postive	Pf					
Eastern	Kaliro	Moderate	1.01711	33.41058	Female	2	F730	2	Positive	Positive	13320	Postive	Pf					
Eastern	Mbale	Low	1.06919	34.20425	Female	9	G383	2	Positive	Positive	1120	Postive	Pf					
Western	Hoima	Low	1.24794	30.81617	Female	3	K381	2	Positive	Positive	7160	Postive	Pf					
Western	Ntungamo	Low	-0.9241	30.45942	Male	10	N849	2	Positive	Positive	176	Postive	Pf					
Western	Ntoroko	Low	1.05278	30.53763	Female	7	C702	2	Positive	Positive	3240	Postive	Pf					
Western	Kamwengye	Low	0.15096	30.43585	Male	10	N304	2	Positive	Positive	47480	Postive	Pf					
Eastern	Mayuge	Moderate	0.47335	33.48334	Female	3	F430	2	Positive	Positive	16	Postive	Pf					
Eastern	Mayuge	Moderate	0.41658	33.48012	Male	3	F320	2	Positive	Positive	109160	Postive	Pf					
Eastern	Mbale	Low	1.11249	34.2552	Male	10	G084	2	Positive	Positive	1760	Postive	Pf					
Western	Kiruhura	Low	0.18238	30.77905	Male	5	J197	2	Positive	Positive	48	Postive	Pf					
Western	Bundibugyo	Low	0.67315	30.01224	Male	7	G774	2	Positive	Positive	25200	Postive	Pf					
Western	Kisoro	Low	-1.1155	29.65096	Female	9	R303	2	Positive	Positive	640	Postive	Pf					
Eastern	Soroti	Low	1.63359	33.57855	Female	7	A527	2	Positive	Positive	39560	Postive	Pf					
Western	Hoima	Low	1.46735	31.32048	Female	4	K469	2	Positive	Positive	2080	Postive	Pf					
Eastern	Namayingo	Moderate	0.24858	33.88629	Male	3	C873	1	Positive	Positive	56504	Postive	Pf					
Western	Kasese	Low	0.69666	30.44282	Male	10	P707	2	Positive	Positive	47040	Postive	Pf					
Eastern	Mayuge	Low	1.02385	31.45147	Female	9	Q403	2	Positive	Positive	47	Postive	Pf		1			
Eastern	Mayuge	Moderate	0.62044	33.61855	Male	8	E225	2	Positive	Positive	840	Postive	Pf					
